# An investigation of semantic similarity judgments about action and non-action verbs in Parkinson's disease: implications for the Embodied Cognition Framework

**DOI:** 10.3389/fnhum.2013.00146

**Published:** 2013-04-18

**Authors:** David Kemmerer, Luke Miller, Megan K. MacPherson, Jessica Huber, Daniel Tranel

**Affiliations:** ^1^Department of Speech, Language, and Hearing Sciences, Purdue UniversityWest Lafayette, IN, USA; ^2^Department of Psychological Sciences, Purdue UniversityWest Lafayette, IN, USA; ^3^Division of Cognitive Neuroscience, Department of Neurology, University of IowaIowa city, IA, USA; ^4^Department of Cognitive Science, University of CaliforniaSan Diego, CA, USA

**Keywords:** verbs, action, Parkinson disease, mirror neuron system, embodied cognition, mental simulation

## Abstract

The Embodied Cognition Framework maintains that understanding actions requires motor simulations subserved in part by premotor and primary motor regions. This hypothesis predicts that disturbances to these regions should impair comprehension of action verbs but not non-action verbs. We evaluated the performances of 10 patients with Parkinson's disease (PD) and 10 normal comparison (NC) participants on a semantic similarity judgment task (SSJT) that included four classes of action verbs and two classes of non-action verbs. The patients were tested both ON and OFF medication. The most salient results involved the accuracies and reaction times (RTs) for the action verbs taken as a whole and the non-action verbs taken as a whole. With respect to accuracies, the patients did not perform significantly worse than the NC participants for either the action verbs or the non-action verbs, regardless of whether they were ON or OFF their medication. And with respect to RTs, although the patients' responses were significantly slower than those of the NC participants for the action verbs, comparable processing delays were also observed for the non-action verbs; moreover, there was again no notable influence of medication. The major dissociation was therefore not between action and non-action verbs, but rather between accuracies (relatively intact) and RTs (relatively delayed). Overall, the data suggest that semantic similarity judgments for both action and non-action verbs are correct but slow in individuals with PD. These results provide new insights about language processing in PD, and they raise important questions about the explanatory scope of the Embodied Cognition Framework.

## Introduction

In recent years, a great deal of research on the neural substrates of semantics has focused on theoretical and empirical issues surrounding the Embodied Cognition Framework, also known as the Grounded Cognition Framework or the Simulation Framework (for overviews see Gibbs, 2006; Barsalou, 2008; Semin and Smith, 2008; Coello and Bartolo, 2012). The central tenet of this theory is that conceptual knowledge is not purely amodal in format, but is instead anchored in modality-specific input/output systems, such that many forms of semantic processing involve transient re-enactments of various sensorimotor and affective states. When we interact with the world, complex unimodal (e.g., visual) feature patterns that are common across different presentations of the same category of stimuli are captured by conjunctive units in correspondingly unimodal memory systems, and correlations between feature patterns across different modalities (e.g., visual and auditory) are captured by higher-order conjunctive units in more integrative crossmodal memory systems. Conceptual tasks, such as processing word meanings, are assumed to involve partial re-enactments of the sensorimotor and affective states that occurred when the referents were directly experienced. According to the Embodied Cognition Framework, these recapitulations or simulations are modality-specific in format. However, because they are driven in top-down rather than bottom-up fashion, they are modulated by many task-specific factors, are rarely represented as complete images, and are not necessarily conscious. Not surprisingly, this theory is quite controversial. It has, however, received support from many sources, including studies which suggest that the comprehension of nouns for concrete entities involves the rapid activation of cortically distributed, modality-specific representations of object properties such as shape (e.g., Wheatley et al., 2005), color (e.g., Simmons et al., 2007), sound (e.g., Kiefer et al., 2008), smell (e.g., González et al., 2006), taste (e.g., Barrós-Loscertales et al., 2012), and manipulability (e.g., Hoenig et al., 2008).

Within the Embodied Cognition Framework, there has been growing interest in the domain of action concepts. One particular question that has been attracting increasing attention is whether comprehending an action verb involves simulating the kind of action to which it refers, using some of the same brain structures that underlie the execution of that action. More precisely, the question is this: are the body-part-specific motor features of the meanings of action verbs—e.g., the types of lip/tongue, arm/hand, and leg/foot actions designated by *lick, pick*, and *kick*, respectively—subserved by the corresponding body-part-specific regions of the left primary motor and/or premotor cortices? In accord with the Embodied Cognition Framework, numerous studies employing diverse brain mapping methods suggest that reading or hearing action verbs does in fact elicit motor activations that are somatotopically mapped, rapidly triggered, and functionally relevant to comprehension (for reviews see Pulvermüller, 2005, 2008; Willems and Hagoort, 2007; Fischer and Zwaan, 2008; Hauk et al., 2008; Fernandino and Iacoboni, 2010; Kemmerer and Gonzalez-Castillo, 2010; Coello and Bartolo, 2012).

At the same time, however, there are also reasons to suppose that motor simulation during the comprehension of action verbs, as well as during the recognition of directly perceived actions, is not an all-or-nothing affair, but is instead an experientially dependent, situationally variable phenomenon (Taylor and Zwaan, 2009; Willems and Casasanto, 2011). For example, a recent fMRI study showed that handedness significantly influences the hemispheric asymmetry of cortical activation patterns when subjects process manual action verbs, such that right-handers engage predominantly left-lateralized hand-related premotor areas, whereas left-handers engage predominantly right-lateralized hand-related premotor areas (Willems et al., 2010; for related data on action observation see Willems and Hagoort, 2009). Focusing on a much more specific kind of expertise, another recent fMRI study demonstrated that skilled hockey players not only understood sentences about hockey maneuvers better than novices, but also exhibited greater activation in the left dorsal premotor cortex while processing such sentences (Beilock et al., 2008; see also Lyons et al., 2010). Several other brain mapping studies have reported similar expertise effects in non-linguistic action recognition, essentially showing that greater skill at executing certain kinds of actions correlates with greater engagement of body-part-congruent frontal motor regions when those kinds of actions are perceived (Calvo-Merino et al., 2006; Cross et al., 2006, 2009; Aglioti et al., 2008; Van Elk et al., 2008; Candidi et al., 2013).

Of all the unresolved questions in this field of inquiry, perhaps the most important is the following: Under what conditions is motor simulation actually necessary for understanding linguistically represented and/or directly perceived actions? A few studies have provided some hints that damage to motor-related regions of the frontal lobes does cause deficits affecting semantic aspects of action verbs (Kemmerer and Tranel, 2003; Neininger and Pulvermüller, 2003; Bak and Hodges, 2004; Hillis et al., 2004, 2006; Grossman et al., 2008; Kemmerer et al., 2012). To take just one example, in a study involving 34 patients with amyotrophic lateral sclerosis, Grossman et al. (2008) found that atrophy in the motor cortex significantly disrupted comprehension of action verbs but not object nouns. Conversely, several investigations have generated results that appear to challenge the Embodied Cognition Framework. For instance, Arévalo et al. (2012) conducted an experiment in which 27 patients with left-hemisphere strokes were given a task that required them to judge whether a given word correctly described a picture of an action involving face-related, arm/hand-related, or leg/foot-related movement. Many of the patients had lesions that included frontal motor areas, but contrary to the predictions of the theory, significant correlations were not found between impaired performance on specific body-part-related action categories and damage to the corresponding body-part-related motor areas. In another notable study, Papeo et al. (2010) asked 12 patients with left-hemisphere strokes to not only imitate pantomimes of certain actions, but also produce and comprehend the verbs that designate them. Challenging the theory once again, double dissociations were observed between the imitation and verb processing tasks. Of greatest relevance in the current context are a few patients who could no longer imitate actions accurately, but could nevertheless understand the associated verbs without major difficulty. These results suggest that motor simulations may not always be necessary to appreciate linguistic descriptions of actions (for further discussion see Papeo and Hochmann, 2012).

Conflicting results have also been reported regarding the issue of whether non-linguistic action understanding necessarily requires motor simulation. On the one hand, a few neuropsychological studies suggest that frontally mediated motor simulation may in fact be essential for the proper recognition of visually perceived actions (Tranel et al., 2003; Saygin et al., 2004; Saygin, 2007; Serino et al., 2009; Kemmerer et al., 2012). In this context, two recent studies by Pazzaglia et al. (2008a,b) are especially noteworthy, since they indicate that some brain-damaged patients with limb apraxia have parallel production and recognition impairments for actions involving tool use, with strong deficit-lesion associations that are selective for particular action categories and particular frontal regions. On the other hand, it has also been shown that some apraxic patients have impaired knowledge of how to use tools, but can nevertheless discriminate between correct and incorrect uses of tools when they see the objects being manipulated by other people (e.g., Halsband et al., 2001; Rumiati et al., 2001; Negri et al., 2007; for theoretical discussion see Mahon and Caramazza, 2005, 2008). And in a similar vein, although rhesus monkeys are biomechanically incapable of throwing objects in an overhand manner, they can nevertheless predict quite accurately the outcomes of overhand throwing actions that they see humans perform (Wood et al., 2007; see also Wood and Hauser, 2008).

One potentially fruitful way to shed more light on the role(s) that frontal motor areas play in action verb comprehension would be to study patients with Parkinson's disease (PD), a degenerative movement disorder characterized mainly by akinesia, bradykinesia, gait abnormalities, resting tremor, and rigidity. PD is caused by progressive dopamine deficiency in the nigrostriatal pathway (Dauer and Przedborski, 2003; Bartels and Leenders, 2009). Striatal dopamine depletion reduces basal ganglia outflow to frontal motor regions (Alexander et al., 1986; Alexander and Crutcher, 1990), leading to dysregulation of the presupplementary motor area, supplementary motor area, primary motor cortex, and ventral premotor cortex (for a review of functional neuroimaging studies, see Grafton, 2004). The literature has yielded partly conflicting results regarding the exact nature of the altered activation levels in these motor cortices during movement execution; however, the most common pattern appears to be the following: (1) hypoactivation in the presupplementary motor area, supplementary motor area, and primary motor cortex (Jenkins et al., 1992; Playford et al., 1992; Rascol et al., 1992; Jahanshahi et al., 1995; Sabatini et al., 2000; Buhmann et al., 2003); and (2) hyperactivation in the ventral premotor cortex (Samuel et al., 1997; Catalan et al., 1999; Hanakawa et al., 1999; Sabatini et al., 2000), perhaps reflecting a compensatory mechanism (Sabatini et al., 2000; Rothwell and Huang, 2003). Relatively normal levels of activation in all of these cortical regions can be restored, however, by levodopa (L-DOPA) treatment (Dick et al., 1987; Ridding et al., 1995; Haslinger et al., 2001; Pierantozzi et al., 2001).

The relevance of PD to current research on the neural substrates of action verbs, and to the Embodied Cognition Framework more broadly, is as follows. If, as the strong version of the theory maintains, the motor features of the meanings of action verbs rely on left frontal motor regions, then one might expect the processing of those semantic features to be affected by the dysregulation of those cortical regions that occurs in PD. Guided by such reasoning, Boulenger et al. (2008) recently investigated how non-demented PD patients both ON and OFF their medication performed on a lexical decision task in a masked repetition priming paradigm. On each trial, participants were first shown a masked stimulus for 50 ms. Then, 100 ms later, they were shown a letter string which they had to judge as being either a real word or a non-word. The real words were either action verbs or object nouns, and the masked stimuli were either consonant strings or the same real words that were used for lexical decision. In the OFF condition, the patients' responses to nouns were significantly faster when the masked stimuli were the very same nouns, compared to when they were consonant strings; however, the patients' responses to verbs were not significantly faster when the masked stimuli were the very same verbs, compared to when they were consonant strings. In the ON condition, significant priming effects were found for both nouns and verbs. The authors argue that their study supports the Embodied Cognition Framework, claiming specifically that the results provide “compelling evidence that processing lexico-semantic information about action words depends on the integrity of the motor system” (Boulenger et al., 2008, p. 743).

Boulenger et al.'s (2008) study is not without shortcomings, however. First, Mahon and Caramazza (2008) point out that in the OFF condition relative to the ON condition, the difference between the patients' average response time for nouns and their average response time for verbs was only substantial when the masked stimuli were consonant strings; it was miniscule when the masked stimuli were identical words. According to Mahon and Caramazza (2008), this is problematic because “on the view that the observed interaction is driven by ‘deviant’ semantic processing, the expectation would be for the interaction to be carried by modulations in the identity condition, rather than the consonant string baseline condition” (p. 65). Second, even if that expectation had been borne out, such a result would not necessarily have constituted evidence for the Embodied Cognition Framework. This is because all of the verbs in the study encoded actions and all of the nouns encoded objects, making it impossible to reliably distinguish between semantic category effects and grammatical category effects.

A recent study investigating action verb comprehension in PD patients corrected for the aforementioned confounds present in Boulenger et al.'s (2008) study. Fernandino et al. (2013) administered a semantic similarity judgment task (SSJT) to non-demented PD patients and age-matched healthy controls. The majority of PD patients (17 out of 20) were ON dopaminergic medication at the time of testing. Action verbs as well as abstract verbs were organized into 40 triads for each verb type, and each triad was presented in a triangular arrangement. Subjects made judgments about which of the two verbs at the base of the arrangement was most similar in meaning to the verb at the top. Whereas no differences were found in the profiles of reaction times (RTs) between the two groups of subjects, significant differences did emerge between their accuracies. The healthy controls were equally accurate at judging action verbs and abstract verbs, but the PD patients were significantly less accurate at judging action verbs than abstract verbs. At first glance, these findings appear to confirm one of the predictions made by the Embodied Cognition Framework—specifically, that PD patients should be impaired at processing action verbs but not abstract verbs. However, there are several problems with the researchers' analyses that warrant caution when interpreting their results this way.

According to the Embodied Cognition Framework, patients with PD should be worse at comprehending action verbs *compared* to subjects without a motor impairment. This requires an analysis *between* the different groups (PD and healthy controls), namely a demonstration that there is an interaction between group type and verb type. However, Fernandino et al.'s (2013) analyses were confined almost entirely to within-group *t*-tests that can only expose differences in processing each verb type *within* a group. While an independent samples *t*-test was performed on the verb type accuracy differences between each group, this is an unconventional method for demonstrating an interaction. Furthermore, while a significant difference between each group was found (*p* = 0.031, one-tailed), it is unclear whether this difference was due to a very slight deficit in action verb comprehension (PD mean: 95.5%, control mean: 96.7%) or a very slight facilitation in abstract verb comprehension (PD mean: 97.5%, control mean: 96.9%). This can only be determined by using alternative between-group tests, which were not performed. It is also worth noting that although the researchers did not find a significant difference in RT between the two groups, this too was based on an independent samples *t*-test. Alternative between-group tests might have led to different outcomes, since the data indicate that the PD patients required considerably more time than the control subjects to make their judgments for both action verbs (PD mean: 2451 ms, control mean: 2022 ms) and abstract verbs (PD mean: 2332 ms, control mean: 1890 ms).

The purpose of the present study was to explore in greater detail the question of whether PD affects the semantic processing of action verbs. To that end, we employed a modified version of a task that was used in a recent fMRI study (Kemmerer et al., 2008). That study tested several predictions, all derived from the Embodied Cognition Framework, about the neural correlates of subtle conceptual distinctions between verbs belonging to the following five classes, each defined in terms of both semantic and syntactic properties (Levin, 1993): Running (e.g., *run, jog, walk*), Hitting (e.g., *hit, poke, jab*), Cutting (e.g., *cut, slice, hack*), Speaking (e.g., *yell, whine, whisper*), and Change of State (e.g., *bloom, blossom, wilt*). The main task was called the SSJT, and, as in Fernandino et al.'s (2013) investigation, it involved making fine-grained discriminations among triads of verbs within each class (e.g., determining that *trudge* is more like *limp* than *stroll*, that *pound* is more like *pummel* than *prod*, that *hack* is more like *chop* than *carve*, etc.), and the baseline task involved making comparable judgments about strings of characters in Wingdings font. Contrary to the authors' expectations, and also contrary to the previous fMRI studies by Tettamanti et al. (2005) and Aziz-Zadeh et al. (2006), Speaking verbs did not engage any lip/tongue-related motor regions^1^. However, in keeping with the Embodied Cognition Framework, Running verbs engaged a putatively leg/foot-related left primary motor region, Hitting verbs engaged a putatively arm/hand-related left primary motor region, Cutting verbs engaged a putatively arm/hand-related (and tool-related; see Lewis, 2006) left premotor region, and Change of State verbs did not engage any left primary motor or premotor regions, which was exactly as predicted, since they do not necessarily encode bodily actions^2^.

In the current study, we administered a slightly different version of the SSJT to 10 non-demented PD patients and 10 age- and education-matched normal comparison (NC) participants. In particular, this version of the task included a sixth verb class—namely, so-called Psych verbs (e.g., *amuse, delight, startle;* see Levin, 1993, pp. 188–193). The task therefore consisted of four classes of action verbs—Running, Hitting, Cutting, and Speaking—and two classes of non-action verbs—Change of State and Psych. The PD patients were tested both ON and OFF their dopaminergic treatment.

At the outset of our study, we made the following predictions based on the strong form of the Embodied Cognition Framework—that is, the form which maintains that motor simulations are essential for understanding actions. Relative to the NC participants, when the PD patients are OFF their medication they should exhibit significantly lower accuracies and/or significantly longer response times for the four classes of action verbs, but the two groups should not perform differently for the two classes of non-action verbs. In addition, the patients' performance on action verbs should improve when they are ON their medication, due to the increase in dopamine in the nigrostriatial pathway and the corresponding improvement in the functional afferentation of motor-related left frontal regions. Our primary goal was to test these predictions that derive from the strong form of the Embodied Cognition Framework^3^. In interpreting our results, however, we also took into consideration a weaker form of the Embodied Cognition Framework—that is, a form which maintains that, as suggested by some of the literature reviewed above, although motor simulations can deepen or enrich the understanding of actions, they are not always necessary for such understanding (Binder and Desai, 2011; Meteyard et al., 2012). We return to these issues in the Discussion.

## Methods

### Participants

The PD patients were 10 individuals with the following demographic characteristics: age (*M* = 75.5, *SD* = 6.3); education (*M* = 16.3, *SD* = 3.7); sex (5 male, 5 female); racial composition (100% white). All were right-handed as measured by the Geschwind–Oldfield Questionnaire (*M* = +99.0, *SD* = 2.0), were native speakers of English, and reported no history of neurological or psychiatric illness other than PD. Additional clinical features of the patients are shown in Table 1. They had been diagnosed with PD between 4 and 13 years prior to their participation in this study (*M* = 7.6, *SD* = 2.8), and were undergoing levodopa therapy (*M* = 475 mg/day, *SD* = 175). Although motor disability is often assessed with the Unified Parkinson's Disease Rating Scale (Fahn and Elton, 1987), we were unable to obtain such data for our patients because their neurologists do not routinely use that method of evaluation. We therefore relied on the less complex but still informative Hoehn and Yahr (1967) system for determining each patient's stage of PD (*M* = 2.8, *SD* = 0.4). In addition, we used the Beck Depression Inventory (Beck et al., 1961) to assess each patient's mood (*M* = 13.3, *SD* = 7.5).

**Table 1:** **Demographic and clinical details for PD patients**.

**Patients**	**Age (years)**	**Education (years)**	**Sex**	**Duration of PD (years)**	**H&Y stage**	**L-DOPA dose (mg/day)**	**BDI**
PD1	73	14	F	13	3	600	14
PD2	68	16	F	5	3	300 (m)	19
PD3	75	14	F	6	2	300	24
PD4	82	20	M	6	3	400	2
PD5	85	12	M	7	3	300 (a)	3
PD6	73	14	M	9	n.a.	650	13
PD7	70	22	M	10	2	800	17
PD8	77	18	F	10	3	400 (m,b)	12
PD9	84	21	M	4	3	600	7
PD10	68	12	F	6	3	400 (a)	22
Mean (*SD*)	75.5 (6.3)	16.3 (3.7)	–	7.6 (2.8)	2.8 (0.4)	475 (175)	13.3 (7.5)

H&Y, Hoehn and Yahr stage; BDI, Beck Depression Inventory; F, female; M, male; n.a., not available; m, plus Mirapex; a, plus amantadine; b, plus bromocriptine.

To ensure that all of the patients were non-demented and had adequate cognitive function to support performance on the verb processing task described below, the Cognitive Linguistic Quick Test (CLQT; Helm-Estabrooks, 2001) was administered. It screens an individual's mental capacities in the domains of attention, memory, executive function, language, and visuospatial skills, and it provides a “composite” measure of overall cognitive function; in addition, it includes a clock drawing task. For each separate domain, as well as for the composite measure and the clock drawing task, scores are interpreted as indicating one of four levels of severity: within normal limits, mildy impaired, moderately impaired, or severely impaired. We established the following exclusionary criteria for participation in our study. No patient could be classified as more than mildly impaired on the composite measure or the clock drawing task; furthermore, no patient could be classified as severely impaired in any of the separate cognitive domains. Based on these criteria, two patients were excluded from the study prior to forming the final group of 10 patients. While our exclusionary criteria are admittedly somewhat arbitrary, we suspect that no approach is perfect, and the particular method we employed was sufficient for our unique purposes because it allowed us to be confident that all of the patients who we ultimately selected were fully capable of understanding and following the instructions for the verb task. The CLQT results for each of the 10 patients are shown in Table 2.

**Table 2:** **Cognitive Linguistic Quick Test (CLQT) results for PD patients**.

**Patients**	**Attention**	**Memory**	**Executive functions**	**Language**	**Visuospatial skills**	**Composite severity**	**Clock drawing**
PD1	WNL	WNL	WNL	WNL	WNL	WNL	WNL
PD2	WNL	WNL	WNL	WNL	WNL	WNL	WNL
PD3	WNL	WNL	WNL	WNL	WNL	WNL	WNL
PD4	Mild	WNL	Mild	WNL	WNL	WNL	WNL
PD5	WNL	WNL	Mild	WNL	WNL	WNL	WNL
PD6	Mild	WNL	Mild	WNL	Mild	Mild	Mild
PD7	WNL	Mild	WNL	WNL	WNL	WNL	WNL
PD8	WNL	WNL	WNL	WNL	WNL	WNL	WNL
PD9	Mild	Mild	Mild	WNL	WNL	WNL	WNL
PD10	Moderate	WNL	Mild	WNL	Moderate	Mild	Mild

WNL, within normal limits; Mild, mildly impaired; Moderate, moderately impaired; Severe, severely impaired.

A group of NC participants was also studied. These were 10 native English speakers, selected so as to be free of neurological or psychiatric illness yet closely matched with the PD patients in terms of both age and education. They had the following demographic characteristics: age (*M* = 71.5, *SD* = 9.6); education (*M* = 16.5, *SD* = 3.4); sex (6 male, 4 female); racial composition (100% white). Nine of the participants were fully right-handed (+100), and one was predominantly left-handed (−70), as measured by the Geschwind–Oldfield Questionnaire.

All of the PD patients and NC participants gave written informed consent in accordance with the Human Subjects Committee of Purdue University and federal regulations. They enrolled in the study on a voluntary basis and were financially compensated for their time.

### Materials

All of the participants performed the SSJT. It requires the participant to compare relatively subtle aspects of the meanings of verbs. Each item consists of three verbs in a triangular array—one at the top and two at the bottom—and the task is to indicate, as quickly and accurately as possible, which of the two bottom verbs is more similar in meaning to the one on top. For example: 
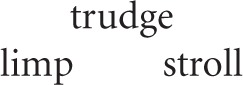
 For each item, all three verbs come from the same semantic class, and the “odd one out” is only moderately different from the other two, so performing the task requires the participant to think carefully about how the verbs relate to each other.

The SSJT contains a total of 144 items—24 from each of the six classes mentioned in the Introduction, namely Running, Hitting, Cutting, Speaking, Change of State, and Psych (for details concerning these verb classes, see Levin, 1993). As noted by Kemmerer et al. (2008), the verbs comprising the items based on the first five classes are not significantly different with respect to either frequency (*M* = 44.9, *SD* = 8.0, *p* = 0.24, frequency data drawn from Carroll et al., 1971) or letter length (*M* = 5.0, *SD* = 1.2, *p* = 0.14). The verbs comprising the items in the Psych condition are closely matched with the verbs comprising the items in the other conditions in terms of frequency (*M* = 43.4, *SD* = 5.8), but they are somewhat longer in terms of letters (*M* = 6.9, *SD* = 1.3).

### Procedures

The SSJT was administered to each participant in 4 separate runs. Each run lasted 4 min and 54 s and contained 6 blocks of items from the SSJT. At the beginning of each block, the word “Verbs” was presented for 5 s followed by 1 s of blank screen. Then 6 items from the SSJT were presented, with each item being shown for 5 s followed by 1 s of blank screen. The verbs comprising the 6 items within a given block were all from the same class (e.g., 6 consecutive items involving Cutting verbs). Each of the 6 classes was represented by 1 block in each run, but the order of class-specific blocks varied across the 4 runs in an unpredictable way. The 6 blocks in each run were separated from each other by 6-s periods during which the participant viewed a flashing fixation cross. In addition, each run began and ended with a 6-s period during which the participant viewed a flashing fixation cross. A complete list of the items is provided in the Appendix.

The SSJT was administered via a laptop computer, and stimulus presentation and response collection were controlled using MacStim (http://www.brainmapping.org/WhiteAnt). The participants responded to each item either by pushing the “m” key with the right index finger to indicate that the verb on the right side of the triangular array was more similar to the one on top, or by pushing the “v” key with the left index finger to indicate that the verb on the left side of the triangular array was more similar to the one on top.

PD patients one through nine were visited at their homes on three separate occasions. (The scheduling of visits for the tenth patient is described below.) On the first visit, each patient received just one run of the SSJT while ON his or her medication. This was done both to familiarize the patient with the task and to obtain an initial baseline measure of performance. The CLQT, Beck Depression Inventory, and Geschwind–Oldfield Questionnaire were administered during the first visit as well, with the following exceptions: the fifth patient (PD5) received the CLQT 15 days prior to the first visit; the sixth patient (PD6) received the CLQT 56 days prior to the first visit; the eighth patient (PD8) received the CLQT 15 days after the first visit; and the ninth patient (PD9) received the CLQT 248 days prior to the first visit. On the second and third visits, each patient received the entire SSJT. The single run of the SSJT that the patient received during the first visit was always the last of the four runs that he or she received during the second and third visits. Moreover, during the second and third visits, the patient received the same sequence of four runs. However, over the course of the study, we employed a Latin-square design such that PD1 received run sequence 1,2,3,4, PD2 received run sequence 2,3,4,1, PD3 received run sequence 3,4,1,2, and so on. One half of the patients were ON their medication during the second visit and OFF it (for at least 12 h) during the third visit, whereas the other half were OFF their medication during the second visit and ON it during the third visit. Across patients one through nine, the first and second visits were separated by an average of 14.3 days (range = 2–44, *SD* = 12.9), and the second and third visits were separated by an average of 19.9 days (range = 14–30, *SD* = 7.1). On each of the three visits, the patients received a practice block of six items before receiving the SSJT. None of the items in this practice block was also included in the SSJT. Finally, with regard to the tenth patient (PD10), she was only visited twice at her home. She was ON her medication during the first visit, and received the entire SSJT as well as the CLQT, Beck Depression Inventory, and Geschwind–Oldfield Questionnaire. She was OFF her medication during the second visit (19 days later), and received the entire SSJT again.

## Results

### Excluded trials

Some participants failed to respond to certain items in the SSJT within the allotted 5-s period. These trials were excluded from the analyses of accuracy and RT presented below. Table 3 indicates the number and proportion of such trials in each verb class for the NC participants, the PD patients in the ON condition, and the PD patients in the OFF condition. Although very few trials were excluded, a *t*-test revealed that the PD patients failed to respond to significantly more items in the OFF condition than in the ON condition (*p* < 0.05).

**Table 3:** **Number (and proportion) of trials in the Semantic Similarity Judgment Task (SSJT) to which participants failed to respond within the allotted 5-s period**.

	**Action verbs**				**Non-action verbs**	
	**Running**	**Hitting**	**Cutting**	**Speaking**	**Change state**	**Psych**
NC	2 (0.8%)	1 (0.4%)	0	0	0	1 (0.4%)
PD ON	7 (2.9%)	1 (0.4%)	9 (3.8%)	2 (0.8%)	3 (1.3%)	5 (2.1%)
PD OFF	6 (2.5%)	5 (2.1%)	10 (4.2%)	5 (2.1%)	7 (2.9%)	5 (2.1%)

These trials were excluded from the analyses of accuracy and reaction time. Note that these trials were subtracted from a total set of 240 for each verb class and each group/condition (24 items per verb class in the SSJT multiplied by 10 participants). NC, normal comparison participants; PD ON, PD patients ON medication; PD OFF, PD patients OFF medication.

### Accuracies

**Figure 1:**
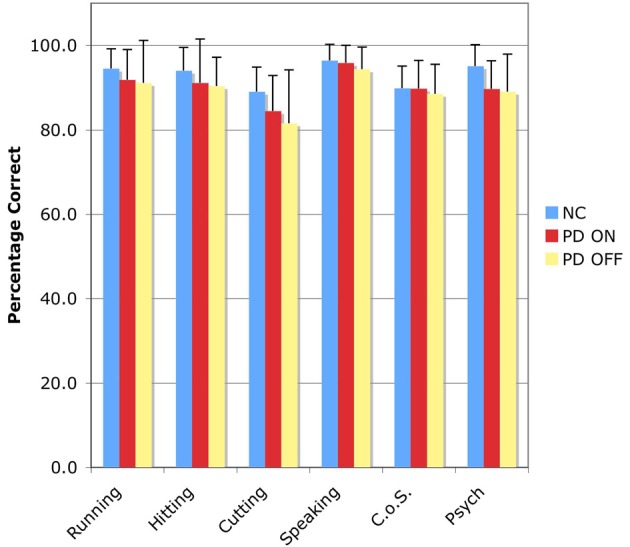
**Accuracy results for the Semantic Similarity Judgment Task (SSJT).** Verb classes are plotted on the horizontal axis, and percent correct is plotted on the vertical axis. Bars represent means and standard deviations. C.o.S., Change of State; NC, normal comparison participants; PD ON, PD patients ON medication; PD OFF, PD patients OFF medication.

The accuracy results for the SSJT are shown in Table 4 and Figure 1.

**Table 4:** **Accuracy results for the Semantic Similarity Judgment Task (SSJT)**.

	**Action verbs**								**Non-action verbs**			
	**Running**		**Hitting**		**Cutting**		**Speaking**		**Change state**		**Psych**	
NC1	100		100		77.8		95.8		88.9		82.6	
NC2	87.5		87.5		95.8		100		87.0		95.7	
NC3	95.8		95.8		91.7		87.5		78.3		95.8	
NC4	87		83.3		87.5		95.8		91.7		100	
NC5	95.8		95.8		91.7		100		91.7		95.7	
NC6	100		100		95.8		95.7		95.8		100	
NC7	95.7		95.8		83.3		100		95.8		95.8	
NC8	91.7		91.7		87.5		87.5		87.5		91.7	
NC9	87.5		95.8		87.5		95.8		95.8		87.5	
NC10	86.4		87.5		79.2		90.9		86.4		87.5	
*M*	92.7		93.3		87.8		94.9		89.9		93.2	
*SD*	5.4		5.6		6.3		4.8		5.5		5.8	
*ME*	±3.35		±3.47		±3.9		±2.98		±3.41		±3.59	
	**ON**	**OFF**	**ON**	**OFF**	**ON**	**OFF**	**ON**	**OFF**	**ON**	**OFF**	**ON**	**OFF**
PD1	91.7	95.7	95.8	83.3	83.3	52.4	91.7	91.7	95.8	95.7	87.5	68.2
PD2	95.8	100	100	100	95.8	91.7	87.5	100	91.7	83.3	91.7	91.7
PD3	95.8	95.8	100	100	87.5	91.7	100	95.8	83.3	91.7	87	91.7
PD4	100	95.7	100	85.7	95	78.3	95.8	95.8	95.7	86.4	95.7	100
PD5	78.3	66.7	78.3	82.6	86.4	87	95.8	95.7	87.5	86.4	95.8	91.7
PD6	81.8	87	70.8	87	70.8	79.2	95.8	82.6	79.2	75	95.8	87.5
PD7	95.7	95.8	95.8	91.7	75	87.5	100	100	87.5	95.8	87	87.5
PD8	91.7	95.8	91.7	95.8	87	75	100	95.7	87.5	87	91.7	95.8
PD9	95.8	87.5	87.5	87.5	79.2	91.3	95.8	91.7	100	95.8	75.0	87.5
PD10	71.4	87.5	95.7	91.7	75.0	86.4	90.9	95.8	95.5	91.3	95.5	90.9
*M*	89.8	90.8	91.6	90.5	83.5	82.1	95.3	94.5	90.4	88.8	90.3	89.3
*SD*	9.4	9.6	9.9	6.4	8.5	12.0	4.2	5.0	6.5	6.6	6.5	8.4
*ME*	±5.83	±5.95	±6.14	±3.97	±5.27	±7.44	±2.6	±3.1	±4.03	±4.09	±4.03	±5.21

Cells indicate percentage correct. NC, normal comparison participants; PD, patients with Parkinson's disease; ON, on medication; OFF, off medication; M, mean; SD, standard deviation; ME, margin of error indicating upper and lower bounds of 95% confidence interval.

#### Action verbs

Three repeated measures analyses of variance (ANOVAs) were used to explore the performance patterns of the NC participants, the PD patients ON medication, and the PD patients OFF medication for the four classes of verbs that collectively fall under the rubric of “action verbs.”

In the first analysis, the between-subjects factor was group—NC vs. PD-ON—and the within-subjects factor was action verb class—Running vs. Hitting vs. Cutting vs. Speaking. There was no effect of group, indicating that the PD patients ON medication did not perform significantly worse than the NC participants. However, there was an effect of action verb class, *F*_(3, 54)_ = 8.873, *p* < 0.001. Follow-up Bonferroni-corrected pairwise comparisons revealed that this effect was driven by significant differences between Cutting verbs and the other three classes of action verbs (all *p*s < 0.05).

In the second analysis, the between-subjects factor was group—NC vs. PD-OFF—and the within-subjects factor was action verb class—Running vs. Hitting vs. Cutting vs. Speaking. There was no effect of group, indicating that the PD patients OFF medication did not perform significantly worse than the NC participants. However, there was again an effect of action verb class, *F*_(3, 54)_ = 8.261, *p* < 0.001. Follow-up Bonferroni-corrected pairwise comparisons identified significant differences between Cutting verbs and two of the other three classes of action verbs, specifically Hitting verbs and Speaking verbs (all *p*s < 0.05).

In the third analysis, the between-subjects factor was group—PD-ON vs. PD-OFF—and the within-subjects factor was action verb class—Running vs. Hitting vs. Cutting vs. Speaking. There was no effect of group, indicating that the PD patients did not perform worse OFF than ON their dopaminergic medication. But once more there was an effect of action verb class. Follow-up Bonferroni-corrected pairwise comparisons pointed again to significant differences between verbs of Cutting and verbs of both Hitting and Speaking (all *p*s < 0.05).

#### Non-action verbs

We also conducted three repeated measures ANOVAs analogous to those described above, only with reference to the two classes of non-action verbs. Across these three analyses, the between-subjects factor was always group, but the particular variables shifted as follows: (1) NC vs. PD-ON; (2) NC vs. PD-OFF; (3) PD-ON vs. PD-OFF. The within-subjects factor was always non-action verb class: Change of State vs. Psych. No significant effects emerged for either factor.

#### Action verbs vs. non-action verbs

Finally, we investigated whether the NC participants, the PD patients in the ON condition, and the PD patients in the OFF condition exhibited significantly different degrees of accuracy on the action verbs taken as a whole compared to the non-action verbs taken as a whole. First we generated for each subject a mean percentage correct score for all four classes of action verbs and another mean percentage correct score for both classes of non-action verbs. This was done twice for the PD patients, once for the ON condition and again for the OFF condition. Then we entered those scores into a repeated measures ANOVA with two factors—group (NC vs. PD-ON vs. PD-OFF) and verb type (action vs. non-action). The analysis revealed no significant effects, indicating that for each of the three groups of interest—namely, NC participants, PD patients ON medication, and PD patients OFF medication—action and non-action verbs elicited comparable levels of accuracy.

### Reaction times

**Figure 2:**
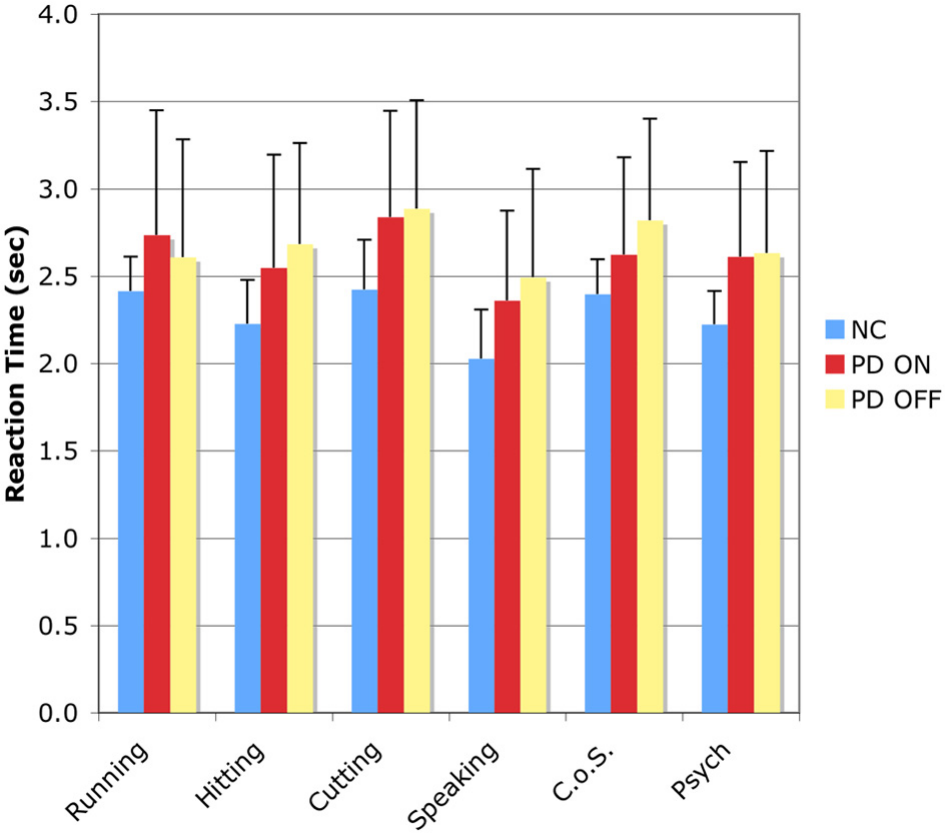
**Reaction time results for the Semantic Similarity Judgment Task (SSJT).** Verb classes are plotted on the horizontal axis, and reaction time in seconds is plotted on the vertical axis. Bars represent means and standard deviations. C.o.S., Change of State; NC, normal comparison participants; PD ON, PD patients ON medication; PD OFF, PD patients OFF medication.

The RT results for the SSJT are shown in Table 5 and Figure 2.

**Table 5:** **Reaction time results for the Semantic Similarity Judgment Task (SSJT)**.

	**Action verbs**								**Non-action verbs**			
	**Running**		**Hitting**		**Cutting**		**Speaking**		**Change state**		**Psych**	
NC1	2.1		2.3		2.7		2.0		2.4		2.3	
NC2	2.7		2.7		2.4		2.0		2.6		2.5	
NC3	2.1		2.2		2.4		1.9		2.3		2.2	
NC4	2.2		2.0		2.2		1.8		2.2		1.9	
NC5	2.3		1.7		1.9		1.7		2.1		1.9	
NC6	2.5		2.5		2.8		2.6		2.8		2.5	
NC7	2.4		2.4		2.7		2.2		2.4		2.2	
NC8	1.7		1.4		1.7		1.7		1.8		1.7	
NC9	2.0		1.9		2.1		2.2		2.3		2.2	
NC10	2.2		2.3		2.3		2.5		2.5		2.2	
*M*	2.2		2.1		2.3		2.1		2.3		2.2	
*SD*	0.3		0.4		0.4		0.3		0.3		0.3	
*ME*	±0.19		±0.25		±0.25		±0.19		±0.19		±0.19	
	**ON**	**OFF**	**ON**	**OFF**	**ON**	**OFF**	**ON**	**OFF**	**ON**	**OFF**	**ON**	**OFF**
PD1	2.8	2.8	2.8	2.9	3.1	2.7	2.5	2.7	2.7	3.0	2.6	3.1
PD2	1.7	1.7	1.5	1.9	1.8	2.0	1.6	1.6	1.6	1.9	1.7	1.8
PD3	2.4	2.0	2.1	2.2	2.3	2.4	1.8	1.7	2.1	2.2	2.2	2.0
PD4	3.0	3.3	2.6	2.7	3.3	3.6	2.7	2.8	2.9	3.4	2.9	2.6
PD5	3.8	3.4	3.4	3.3	3.3	3.5	3.0	3.2	3.0	3.2	3.1	3.0
PD6	3.6	3.1	3.5	3.5	3.5	3.5	3.0	3.3	3.2	3.4	3.2	3.2
PD7	2.7	2.7	2.8	3.0	2.9	3.0	2.4	2.7	2.7	2.9	3.0	3.3
PD8	2.3	2.2	2.2	2.4	2.8	2.7	2.3	2.3	2.8	2.7	2.6	2.2
PD9	1.9	1.9	2.0	2.0	2.2	2.0	1.9	2.0	2.2	2.3	1.9	2.2
PD10	2.9	2.7	3.1	2.4	3.2	2.7	2.4	2.3	3.0	3.0	3.0	2.8
*M*	2.7	2.6	2.6	2.6	2.8	2.8	2.4	2.5	2.6	2.8	2.6	2.6
*SD*	0.7	0.6	0.6	0.5	0.6	0.6	0.5	0.6	0.5	0.5	0.5	0.5
*ME*	±0.43	±0.37	±0.37	±0.31	±0.37	±0.37	±0.31	±0.37	±0.31	±0.31	±0.31	±0.31

Cells indicate reaction time in seconds. NC, normal comparison participants; PD, patients with Parkinson's disease; ON, on medication; OFF, off medication; M, mean; SD, standard deviation; ME, margin of error indicating upper and lower bounds of 95% confidence interval.

#### Action verbs

As before, three repeated measures ANOVAs were used to explore the performance patterns of the NC participants, the PD patients ON medication, and the PD patients OFF medication for the four classes of action verbs.

In the first analysis, the between-subjects factor was group—NC vs. PD-ON—and the within-subjects factor was action verb class—Running vs. Hitting vs. Cutting vs. Speaking. There was an effect of group, *F*_(1, 18)_ = 4.545, *p* < 0.05, indicating that the PD patients in the ON condition responded to the action verbs significantly more slowly than the NC participants. There was also an effect of action verb class, *F*_(3, 54)_ = 14.246, *p* < 0.001, and follow-up Bonferroni-corrected pairwise comparisons revealed significant differences between the following classes (all *p*s < 0.05): Running vs. Speaking; Hitting vs. Cutting; and Cutting vs. Speaking.

In the second analysis, the between-subjects factor was group—NC vs. PD-OFF—and the within-subjects factor was action verb class—Running vs. Hitting vs. Cutting vs. Speaking. Again, there was an effect of group, *F*_(1, 18)_ = 4.575, *p* < 0.05, indicating that the PD patients in the OFF condition responded to the action verbs significantly more slowly than the NC participants. In addition, there was an effect of verb class, *F*_(3, 54)_ = 8.920, *p* < 0.001, and follow-up Bonferroni-corrected pairwise comparisons revealed significant differences between Hitting and Cutting verbs as well as between Cutting and Speaking verbs (all *p*s < 0.05).

In the third analysis, the between-subjects factor was group—PD-ON vs. PD-OFF—and the within-subjects factor was action verb class—Running vs. Hitting vs. Cutting vs. Speaking. No effect of group emerged, indicating that the PD patients were not markedly slower in the OFF than the ON condition. However, an effect of action verb class appeared once more, *F*_(3, 54)_ = 18.685, *p* < 0.001, and follow-up Bonferroni-corrected pairwise comparisons revealed significant differences between all of the classes except Running vs. Hitting (all *p*s < 0.05).

#### Non-action verbs

Another set of repeated measures ANOVAs focused on the RT results pertaining to the two classes of non-action verbs.

In the first analysis, the between-subjects factor was group—NC vs. PD-ON—and the within-subjects factor was non-action verb class—Change of State vs. Psych. There was an effect of group, *F*_(1, 18)_ = 4.225, *p* < 0.05, indicating that the PD patients in the ON condition were significantly slower than the NC participants. In addition, there was an effect of non-action verb class, *F*_(1, 18)_ = 8.679, *p* < 0.01, and follow-up analyses confirmed that response latencies for Change of State verbs were significantly longer than for Psych verbs.

In the second analysis, the between-subjects factor was group—NC vs. PD-OFF—and the within-subjects factor was non-action verb class—Change of State vs. Psych. Again, there was an effect of group, *F*_(1, 18)_ = 2.116, *p* < 0.05, indicating that the PD patients in the OFF condition were slower than the NC participants. Moreover, there was an effect of non-action verb class, *F*_(1, 18)_ = 11.758, *p* < 0.01, with follow-up analyses demonstrating once again that RTs for Change of State verbs were significantly longer than for Psych verbs.

In the third analysis, the between-subjects factor was group—PD-ON vs. PD-OFF—and the within-subjects factor was non-action verb class—Change of State vs. Psych. No significant effects were found.

#### Action verbs vs. non-action verbs

Finally, we investigated whether the NC participants, the PD patients in the ON condition, and the PD patients in the OFF condition displayed significantly different RTs for the action verbs taken as a whole compared to the non-action verbs taken as a whole. As in the treatment of accuracy data described in section Action Verbs vs. Non-Action Verbs, we first generated for each subject a mean RT for all four classes of action verbs and another mean RT for both classes of non-action verbs. This was done twice for the PD patients, once for the ON condition and again for the OFF condition. Then we entered those data into a repeated measures ANOVA with two factors—group (NC vs. PD-ON vs. PD-OFF) and verb type (action vs. non-action). Although the analysis revealed no effect of verb type, it did yield an effect of group, *F*_(1, 2)_ = 4.31, *p* < 0.05. However, none of the follow-up adjusted Tukey-Kramer tests reached significance: NC vs. PD-ON, *p* = 0.098; NC vs. PD-OFF, *p* = 0.059; PD-ON vs. PD-OFF, *p* = 0.981. Overall, the most important finding is that for each of the three groups of interest—namely, NC participants, PD patients ON medication, and PD patients OFF medication—RTs for action verbs were comparable to those for non-action verbs.

### Additional analyses

Given the relatively small samples of subjects in this study, one might argue that non-parametric statistical analyses are more appropriate than parametric ones. For this reason, we also conducted analyses similar to those presented above, only employing Wilcoxon tests. The results of those analyses are consistent with the results of the aforementioned ANOVAs.

In addition, we investigated whether the PD patients' disease durations significantly correlated with their accuracies and/or RTs for the different classes of action and non-action verbs. Regarding accuracies, we did not find any significant correlations when the patients were ON medication; however, we did find two significant correlations when they were OFF medication. Specifically, accuracies for Cutting verbs [r_(9)_ = −0.81, *p* < 0.01] and Psych verbs [r_(9)_ = −0.61, *p* < 0.05] were negatively correlated with disease duration. Thus, longer disease duration led to decreased performance for these verb classes. As for RTs, no significant correlations emerged in either the ON or OFF condition.

## Discussion

In this study we evaluated the ability of 10 non-demented PD patients and 10 NC participants to make fine-grained semantic similarity judgments about four classes of action verbs—Running, Hitting, Cutting, and Speaking—and two classes of non-action verbs—Change of State and Psych. Some interesting effects emerged for one specific class, namely Cutting verbs, and we will briefly consider those findings below. However, the most salient and theoretically relevant results involved the accuracies and RTs for the action verbs taken as a whole and the non-action verbs taken as a whole. With respect to accuracies, the PD patients did not perform significantly worse than the NC participants for either the action verbs or the non-action verbs, regardless of whether they were ON or OFF their dopaminergic medication. And with respect to RTs, although the PD patients' responses were significantly slower than those of the NC participants for the action verbs, comparable processing delays were also observed for the non-action verbs; moreover, there was again no notable influence of medication status. The most pronounced dissociation was therefore not between action and non-action verbs, but rather between accuracies (relatively intact) and RTs (relatively delayed). Overall, the data suggest that semantic similarity judgments for both action and non-action verbs are, for the most part, correct but slow in individuals with PD.

As we pointed out in the Introduction, a similar study was recently reported by Fernandino et al. (2013), and although their statistical analyses had some non-trivial limitations, it is noteworthy that several aspects of their results are comparable to our findings. To briefly reiterate: with respect to accuracies, their patients, like ours, performed at virtually the same level as the healthy control subjects for both action verbs (PD mean: 95.5%, control mean: 96.7%) and abstract verbs (PD mean: 97.5%, control mean: 96.9%). And with respect to RTs, their patients, like ours, took considerably longer than the healthy control subjects to make their judgments for both action verbs (PD mean: 2451 ms, control mean: 2022 ms) and abstract verbs (PD mean: 2332 ms, control mean: 1890 ms).

These behavioral patterns are important not only because they add to the literature on language processing in PD, but also because they are relevant to recent debates surrounding the Embodied Cognition Framework. In what follows, we elaborate several alternative explanations of our results, focusing first on the finding of relatively preserved comprehension of both action and non-action verbs, and then on the finding of relatively delayed semantic processing of both action and non-action verbs. Throughout the discussion, we explore some of the ways in which our study might bear on the Embodied Cognition Framework.

### PD patients have relatively preserved comprehension of both action and non-action verbs

As already noted, the strong form of the Embodied Cognition Framework maintains that understanding actions—both directly perceived and linguistically represented—necessarily requires motor simulations that are mediated in part by left frontal regions, particularly the primary motor and premotor cortices. Because these regions are dysfunctional in PD due to altered afferentation from the basal ganglia, one might suppose that they would no longer be able to support normal motor simulations of the kinds of bodily actions that are typically encoded by verbs. Such a view predicts that PD patients OFF medication would be at least moderately impaired on a task like the SSJT, which forces participants to make subtle semantic similarity judgments about action verbs. We found, however, that when the four classes of action verbs in the SSJT were analyzed as a whole, the PD patients OFF medication performed just as accurately as the control subjects. This discovery therefore seems to pose a challenge to the strong form of the Embodied Cognition Framework.

It is important to recognize, though, that this line of argumentation hinges on the key assumption that the capacity for motor simulation is in fact disrupted in PD. To be sure, there are a few hints that in this population implicit motor simulations are abnormal during the observation of actions. Specifically, two recent studies have shown that, relative to control subjects, PD patients do not exhibit normal corticomotor facilitation (Tremblay et al., 2008) or behavioral facilitation (Castiello et al., 2009) during the observation of actions performed by neurologically healthy adults. In addition, a few studies have revealed abnormalities involving explicit motor imagery in PD patients (Dominey et al., 1995; Cunnington et al., 2001; Thobois et al., 2002; Amick et al., 2006; Helmich et al., 2007). To the best of our knowledge, however, nothing else is currently known about the capacity for motor simulation in PD, and this raises difficult questions about whether it is really possible, at this stage of inquiry, to use the Embodied Cognition Framework to formulate clear predictions regarding the status of verb comprehension in PD.

Several possibilities are worth considering. One is that PD does disrupt motor simulations during verb comprehension, but only to a mild degree, so that such simulations can still help patients determine the semantic relations among the action verbs in the SSJT. This view is still compatible with the strong form of the Embodied Cognition Framework; however, it predicts that PD patients would exhibit lower accuracies on a task that required substantially more attention to the motor features of verb meanings. In addition, it predicts that stroke patients who have suffered direct focal lesions to body-part-specific motor areas would have, relative to PD patients, more severely disrupted capacities for motor simulation, and hence would be more likely to perform poorly on the action verbs in the SSJT. Further research is needed to test these hypotheses.

Yet another possibility is that the capacity for motor simulation is impaired to a non-trivial extent in PD; however, this disturbance is not sufficient to prevent patients from achieving a high level of accuracy on the action verbs in the SSJT. This view cannot easily be reconciled with the strong form of the Embodied Cognition Framework, but it is consistent with a weaker form of the theory which maintains that it is not always necessary to run motor simulations in left frontal regions in order to appreciate the nuances of action verbs; instead, other types of modality-specific semantic representations subserved by other cortical areas may be adequate for many comprehension tasks, including the SSJT (Taylor and Zwaan, 2009). For example, it is noteworthy that in Kemmerer et al.'s (2008) fMRI study, verbs of Running, Hitting, and Cutting engaged not only somatotopically mapped motor areas in the left frontal lobe, but also a number of additional regions, one of which was the left posterolateral temporal cortex (encompassing the posterior superior temporal sulcus and the adjacent posterior middle temporal gyrus), an area that may contribute to representing, at least in a schematic manner, the types of visual motion patterns that are encoded by verbs (see also Kable et al., 2002, 2005; Tranel et al., 2003, 2005, 2008; Deen and McCarthy, 2010; Wallentin et al., 2011; Kemmerer et al., 2012; Humphreys et al., 2013; Peelen et al., 2013; for a review see Gennari, 2012). Importantly, the fact that all of these areas were engaged does not mean that all of them are essential for successful task performance. Indeed, taken by themselves, the fMRI results are compatible with the possibility that healthy individuals—and also, crucially, the PD patients in the current study—might be able to perform fairly well on the SSJT by relying more on visual information represented in the left posterolateral temporal cortex than on motor information represented in the left frontal cortex.

Although this account is internally coherent, its explanatory power is also limited. As we mentioned in the Introduction, there is independent evidence that directly affecting the operations of the left primary motor and/or premotor cortices does, at least in some circumstances, have functional consequences for understanding action verbs. For example, single-pulse TMS applied to hand-related left primary motor cortex facilitates lexical decisions for hand-related verbs but not leg-related verbs, and conversely, stimulation of leg-related left primary motor cortex facilitates lexical decisions for leg-related verbs but not hand-related verbs (Pulvermüller, 2005). In addition, repetitive TMS applied to hand-related left primary motor cortex delays the process of making morphological transformations of both action verbs and action nouns, but does not influence this process for either state verbs or state nouns (Gerfo et al., 2008). Furthermore, a few neuropsychological studies suggest that damage to left motor areas can impair the understanding of not only action verbs (Kemmerer and Tranel, 2003; Bak and Hodges, 2004; Hillis et al., 2004, 2006; Grossman et al., 2008; Kemmerer et al., 2012) but also non-linguistic action concepts (Tranel et al., 2003; Saygin et al., 2004; Saygin, 2007; Pazzaglia et al., 2008a,b; Serino et al., 2009; Kemmerer et al., 2012). Nevertheless, it is also worth recalling from the Introduction that the neuropsychological literature on this topic is somewhat mixed, since some patients with action production deficits can still appreciate the corresponding verbs (Papeo et al., 2010) and/or still visually discriminate between correct and incorrect object-directed movements (e.g., Halsband et al., 2001; Rumiati et al., 2001; Negri et al., 2007).

So far we have been dealing with action verbs in general, but at this juncture it is worth recalling that our study did reveal some relatively small but nevertheless statistically significant accuracy differences between the four classes of action verbs in the SSJT. In particular, Cutting verbs elicited lower scores than the other types of verbs, and performance differences emerged not only between the NC participants and the PD patients, but also between the PD patients in the ON and OFF conditions. Converging with this finding is the additional discovery that the patients' accuracies on Cutting verbs, but not on any of the other types of action verbs, correlated significantly with their disease duration such that the lowest scores were obtained by those patients with the longest histories of PD. In keeping with these results, it is also notable that in Kemmerer et al.'s (2008) fMRI study, Cutting verbs engaged by far the largest cluster of voxels in the left frontal lobe, encompassing portions of the hand-related ventral premotor region that is well-established as being dysfunctional in PD (Samuel et al., 1997; Catalan et al., 1999; Hanakawa et al., 1999; Sabatini et al., 2000). Taken together, these considerations suggest that if we restrict our attention to just this one narrow class of action verbs, the accuracy data can in fact be accommodated by the strong form of the Embodied Cognition Framework. At the same time, however, we would like to emphasize that in the broader context of the study as a whole, this is a fairly minor result that should not be over-interpreted.

More generally, it remains puzzling why the PD patients in the current study manifested relatively intact comprehension of the other three classes of action verbs, and it is hard to determine precisely what this finding implies about the Embodied Cognition Framework. We submit that the correct interpretation is uncertain mainly because of the following two factors, both of which we elaborated above: first, it is not clear how much PD affects the ability of the frontal lobes to support motor simulations during action observation and action verb comprehension; and second, there are different forms of the Embodied Cognition Framework—strong and weak—that make different claims about the functional importance of motor simulations during action observation and action verb comprehension.

Before moving on to discuss the RT results, it may be worthwhile to step back for a moment and take a broader theoretical perspective on the issues surrounding the accuracy data. According to recent research on the neural substrates of semantic knowledge, the meanings of words depend not only on modality-specific brain systems for perception and action, but also on higher-order integrative mechanisms in the anterior temporal lobes (ATLs) that serve to bind and organize the multifarious crossmodal features of concepts (e.g., Simmons and Barsalou, 2003; Patterson et al., 2007; Binney et al., 2010; Lambon Ralph et al., 2010; Visser et al., 2010; Peelen and Caramazza, 2012; note that the left angular gyrus may have similar integrative functions, as suggested by Binder et al., 2009, Bonner et al., 2013 and Seghier, 2013). Although most of this work has focused on object concepts, there is growing evidence that the ATLs also contribute to the representation of action concepts (Cotelli et al., 2006; Hillis et al., 2006; Murray et al., 2007; Pulvermüller et al., 2009) and abstract concepts (Jefferies et al., 2009; Pobric et al., 2009; Wang et al., 2010; Hoffman and Lambon Ralph, 2011). Now, some investigators—see especially the work of Matthew Lambon Ralph and his colleagues—have argued that the semantic representations in the ATLs are completely amodal in character. This proposal has been challenged (Skipper et al., 2011; Gainotti, 2012), but even if we assume, for the sake of argument, that it is correct, we are not necessarily forced to accept a theory that accounts for conceptual processing entirely in terms of amodal representations. Instead, the possibility opens up for a theory that posits rich interactions between amodal representations on the one hand and modality-specific representations on the other, along the lines of the so-called “hub and spoke” model that Lambon Ralph and his colleagues have been developing (e.g., Lambon Ralph et al., 2010; Pobric et al., 2010; Hoffman and Lambon Ralph, 2013). This type of hybrid approach builds on the Embodied Cognition Framework in important ways, and it suggests that the PD patients in the current study may have benefited from having intact amodal representations of verb meanings in the ATL. It is also possible that these amodal representations are accessed rapidly and automatically, whereas the related modality-specific representations are accessed more slowly and strategically, but further research is required to determine whether this is really the case (for theoretical discussion see Mahon and Caramazza, 2008, and Tomasino and Rumiati, 2013; and for related electrophysiological data involving object concepts see Chan et al., 2011 and Naci et al., 2012).

### PD patients have relatively slow semantic processing of both action and non-action verbs

We turn now to the RT results. Based on the strong form of the Embodied Cognition Framework, together with the fact that PD reduces basal ganglia outflow to the frontal lobes and thereby leads to hypoactivation of the majority of motor cortices, one could reasonably predict that PD patients OFF medication would have abnormally long RTs for the action verbs, but not the non-action verbs, in the SSJT. What we found, however, is that the patients' responses were markedly delayed for both of these general categories of verbs. In addition, these delays were not significantly reduced when the patients performed the task while ON medication. These results therefore appear to challenge the strong form of the Embodied Cognition Framework.

One way to explain the RT results, in a manner that would still be compatible with the weak form of the Embodied Cognition Framework, would be to assume that PD prolongs either or both of the following two phases of the comprehension process that is tapped by all of the items, action-related as well as non-action-related, in the SSJT: (1) the initial activation of the idiosyncratic semantic features of particular verbs; and (2) the subsequent analysis and comparison of the semantic features of different verbs through the deliberate use of working memory and attentional control. Regarding phase 1, as indicated in the Introduction, Boulenger et al. (2008) ostensibly demonstrated that immediate semantic activation is more impaired for action verbs than object nouns in PD. However, we pointed out several limitations of that study, and it is noteworthy that several other studies suggest that dopamine and the basal ganglia exert an influence on semantic activation for not just action verbs but also object nouns (Kischka et al., 1996; Copland, 2003; Angwin et al., 2004, 2009; Pederzolli et al., 2008; Copland et al., 2009; see also Crosson et al., 2007). It is therefore conceivable that the patients in our study suffered from delays in initial semantic activation for many kinds of words, and that these delays contributed to their abnormally long response times for both the action verbs and the non-action verbs in the SSJT.

Regarding phase 2 of the comprehension process, it is also possible that the patients' abnormally long response times for both types of verbs reflect delays in carrying out the voluntarily controlled semantic analyses and comparisons that are necessary for explicitly judging the different degrees of similarity among the three verbs comprising each item in the SSJT, regardless of whether those verbs do or do not designate actions. Recent research suggests that semantic working memory depends on certain sectors of the left inferior frontal gyrus, with the pars orbitalis (~BA47) supporting mainly the retrieval of specific semantic structures stored in other brain regions, and the pars triangularis (~BA45) supporting mainly the post-retrieval resolution of competitions among activated representations (for a review see Badre and Wagner, 2007; see also, e.g., Thompson-Schill et al., 1998, 1999; Moss et al., 2005; Gold et al., 2006; Bedny et al., 2008). These left inferior frontal areas were engaged by all of the verb classes in Kemmerer et al.'s (2008) fMRI study, and from the perspective of the Embodied Cognition Framework, they may play important roles in the strategic process of guiding and manipulating simulations of various modality-specific aspects of verb meaning in other cortical regions. Importantly, these areas may be involved in circuits with the basal ganglia (Ullman, 2006), and hence they may be dysfunctional in PD, leading to a general slowing of strategic semantic processing. The hypothesis that PD affects phase 2 of the comprehension process tapped by the SSJT has the additional virtue of converging with a large literature pointing to deficits in working memory and attentional control in PD (e.g., Lees and Smith, 1983; Taylor et al., 1986; Cooper et al., 1991; Gabrieli et al., 1996; Lewis et al., 2003; Moustafa et al., 2008; for a review see Owen, 2004).

Might slowness in the initiation and/or execution of button pressing be another factor contributing to the patients' abnormally long RTs for both action and non-action verbs in the SSJT? This is certainly possible. Unfortunately, we did not include in our experiment an independent measure of the speed of cued button pressing. However, we suspect that even if slowness in this domain were present, it would only account for a relatively small proportion of the patients' response delays when performing the SSJT. For instance, in Boulenger et al.'s (2008) study of lexical decisions in a masked repetition priming paradigm, when PD patients pressed buttons in response to nouns while ON their medication, their RTs were only about 70 ms slower than those of the control subjects, and of course some of that delay could have reflected slowness in the lexical decision process itself, rather than in the planning and/or execution of button pressing. In our study, if one averages across all six classes of verbs, the PD patients ON medication were about 400 ms slower than the NC participants (consistent with the results reported by Fernandino et al., 2013), and the PD patients OFF medication were about 500 ms slower. Thus, while slowness in button pressing may have contributed slightly to the patients' response delays, those delays were most likely due primarily to protracted semantic processing.

Finally, although the PD patients failed to meet the 5-s response time cutoff for significantly more trials in the OFF condition than in the ON condition (see section Excluded Trials), it is noteworthy that for the trials that they did complete, they were not significantly slower at making judgments in the OFF condition than in the ON condition. This outcome goes against our expectation that dopaminergic treatment would significantly facilitate semantic processing in the ON condition. However, while such treatment is known to improve the motor symptoms of PD, its effects on cognition are more complex, and a wide range of positive, negative, and neutral influences have been observed, depending on a variety of factors such as task demands and basal dopamine levels (for a review see Cools, 2006). For example, at least two studies have found that L-DOPA does not change PD patients' performance on the Wisconsin Card Sorting Task (Gotham et al., 1988; Lange et al., 1995). Consequently, one cannot simply assume that cortical activity levels are completely “normal” when patients are ON medication. Our findings suggest that current medications may not be very effective at ameliorating delayed semantic processing in PD. Further investigation will hopefully shed more light on this topic.

### Conclusion

We investigated the ability of PD patients to make subtle semantic similarity judgments about action and non-action verbs. Our results indicate that such judgments are, for the most part, accurate but slow for both types of verbs, regardless of whether the patients are ON or OFF medication. We have interpreted these findings largely in the context of one of the most controversial theories of knowledge representation, namely the Embodied Cognition Framework, which maintains that concepts are grounded in modality-specific input/output systems, such that many forms of semantic processing involve transient re-enactments or simulations of sensory, motor, and affective states. After considering the relevant issues from several perspectives, we have concluded that, at this stage of inquiry, it is very difficult to draw any definitive implications of our findings for the Embodied Cognition Framework because, first, it is not clear to what extent frontally mediated motor simulations are disrupted in PD, and second, there are currently at least two alternative versions of the theory—strong and weak—which differ as to whether motor simulations play an essential or merely augmentative role in action verb comprehension. Nevertheless, it remains the case that the empirical results of our study are novel and valuable, since they contribute substantially to the literature on how language is and is not affected by PD.

### Conflict of interest statement

The authors declare that the research was conducted in the absence of any commercial or financial relationships that could be construed as a potential conflict of interest.

## Footnotes

^1^This could reflect the greater importance of auditory and emotional features, relative to motor features, in the meanings of Speaking verbs.

^2^As indicated by Kemmerer et al. (2008), some of the Change of State verbs in the SSJT encode internally caused object transformations (e.g., *rust*), which clearly have nothing to do with bodily actions. Most of the Change of State verbs in the SSJT, however, encode externally caused object transformations (e.g., *shatter*), and they can optionally specify agentive object-directed movement (e.g., *The glass shattered* alternates with *Bill shattered the glass*; see Levin, 1993, pp. 5–11, 240–248). Nevertheless, even when externally caused Change of State verbs are used transitively, they rarely refer to particular kinds of body-part-specific actions. For all of these reasons, the meanings of Change of State verbs in general are not expected to depend on somatotopically mapped primary motor and/or premotor cortices, unlike verbs of Running, Hitting, Cutting, and Speaking.

^3^One of the reviewers noted that some advocates of the Embodied Cognition Framework maintain that not only concrete concepts but also abstract concepts depend to some extent on the sensorimotor system (e.g., Barsalou and Wiemer-Hastings, 2005; Pecher et al., 2011; Scorolli et al., 2011; Wilson-Mendenhall et al., 2011). This point is well-taken. However, regarding the current study, as indicated above, we already have fMRI data showing that when normal subjects make semantic similarity judgments involving the specific Change of State verbs that we used in our task, somatotopically mapped motor areas are not significantly engaged (Kemmerer et al., 2008). In addition, other fMRI work has shown that the comprehension of sentences encoding mental states/processes does not significantly activate somatotopically mapped motor areas (Tettamanti et al., 2005). Partly for this reason, we would not expect the Psych verbs in our study to rely upon those areas. It is also noteworthy that, like the Change of State verbs, none of the Psych verbs refer to body-part-specific actions.

## Appendix

The matrix below shows the stimuli for the Semantic Similarity Judgment Task (SSJT), organized according to the design of the 6 blocks of verb trials in each of the 4 runs of the experiment. The names of the verb classes are provided only for explanatory purposes; they were not included in the experimental paradigm itself, and the subjects were unable to predict which verb class would be represented in each consecutive block. The block and trial sequences are, however, exactly as they were in the actual experiment. For each trial, the pivot verb that appeared at the top of the triangular array is listed first; that verb is followed by the one that appeared below and to the left of the pivot; and that verb in turn is followed by the one that appeared below and to the right of the pivot. For further details see the Appendix of Kemmerer et al. (2008).

**Table:**

**Run 1**	**Block 1**	**Block 2**	**Block 3**	**Block 4**	**Block 5**	**Block 6**
**Trial**	**Running**	**Speaking**	**Psych**	**Hitting**	**Cutting**	**Change state**
1	walk	yell	amuse	spank	snip	break
	stumble	shout	delight	tap	cut	snap
	amble	whimper	excite	slap	grate	bend
2	stagger	mumble	dismay	stroke	hack	tear
	tiptoe	mutter	frighten	caress	carve	crack
	limp	bawl	sadden	hit	chop	rip
3	run	holler	embarrass	prick	mash	crease
	jog	bellow	humiliate	pat	gash	twist
	sneak	whine	discourage	poke	squish	fold
4	stomp	chatter	disturb	pummel	shred	bloom
	march	stutter	bother	knock	slice	blossom
	saunter	jabber	torment	batter	gouge	sprout
5	stumble	scream	annoy	clobber	slit	chip
	walk	moan	enrage	whack	nick	splinter
	trip	shriek	irritate	prick	mince	smash
6	limp	cry	reassure	tap	grate	twist
	stroll	bawl	comfort	pound	pierce	bend
	trudge	sing	arouse	prod	shred	rip
**Run 2**	**Block 1**	**Block 2**	**Block 3**	**Block 4**	**Block 5**	**Block 6**
**Trial**	**Hitting**	**Running**	**Change state**	**Speaking**	**Cutting**	**Psych**
1	whack	leap	split	mutter	chop	torment
	strike	jump	shatter	grumble	dice	sadden
	prick	strut	crack	chatter	scrape	enrage
2	poke	tiptoe	bend	shout	nick	astound
	whack	trudge	break	whimper	scratch	astonish
	jab	creep	twist	yell	hack	annoy
3	hit	march	wilt	cry	slice	irritate
	tap	stomp	bloom	wail	slash	bother
	knock	amble	wither	mumble	carve	frighten
4	caress	sprint	rip	chant	gouge	comfort
	pat	run	tear	scream	slit	amuse
	clobber	walk	fold	sing	pierce	soothe
5	batter	skip	blossom	groan	clip	delight
	pound	limp	bloom	bawl	cut	inspire
	poke	dance	sprout	whine	squish	thrill
6	slap	jog	break	grumble	mince	depress
	jab	trudge	bend	whimper	gash	disturb
	smack	skip	shatter	holler	chop	dismay
**Run 3**	**Block 1**	**Block 2**	**Block 3**	**Block 4**	**Block 5**	**Block 6**
**Trial**	**Change state**	**Psych**	**Hitting**	**Running**	**Cutting**	**Speaking**
1	smash	frighten	jab	strut	scratch	whimper
	shatter	enrage	hit	traipse	gouge	cry
	twist	scare	poke	limp	clip	bellow
2	sprout	thrill	smack	amble	scrape	shout
	wilt	amuse	stroke	stroll	snip	murmur
	blossom	excite	spank	skip	gash	holler
3	crumble	sadden	pinch	plod	carve	shriek
	break	depress	prick	run	crush	whisper
	crease	frighten	pat	trudge	cut	yell
4	crack	arouse	pound	hop	dice	bellow
	split	inspire	pummel	stumble	chop	shout
	rip	startle	prod	jump	shred	cry
5	shatter	encourage	pat	sashay	cut	whine
	crumple	delight	pound	strut	slice	scream
	smash	reassure	tap	stagger	mash	groan
6	wither	enrage	strike	stroll	gash	murmur
	bloom	bother	poke	sneak	grind	yelp
	wilt	torment	hit	saunter	scratch	mumble
**Run 4**	**Block 1**	**Block 2**	**Block 3**	**Block 4**	**Block 5**	**Block 6**
**Trial**	**Cutting**	**Hitting**	**Speaking**	**Psych**	**Change state**	**Running**
1	slice	prod	mutter	bother	snap	saunter
	cut	tap	chatter	annoy	fold	sashay
	grind	strike	mumble	scare	break	sprint
2	nick	tap	bawl	discourage	crumple	trudge
	slit	clobber	sing	embarrass	bend	jog
	carve	pat	wail	sadden	split	plod
3	scratch	spank	whisper	excite	sprout	sneak
	chop	poke	murmur	astonish	wither	tiptoe
	gash	smack	whine	arouse	bloom	stagger
4	squish	knock	sing	startle	fracture	jump
	slice	whack	chant	surprise	crack	march
	mash	prick	shriek	thrill	shatter	hop
5	slash	pummel	holler	scare	splinter	traipse
	hack	caress	mutter	frighten	crumple	saunter
	nick	batter	shout	sadden	chip	run
6	grind	hit	wail	soothe	fold	walk
	slice	pat	cry	console	crease	jog
	mince	strike	stutter	excite	snap	stroll

## References

[B1] Aglioti S. M. Cesari P. Romani M. Urgesi C. (2008). Action anticipation and motor resonance in elite basketball players. Nat. Neurosci. 11, 1109–1116 19160510 10.1038/nn.2182

[B2] Alexander G. E. Crutcher M. D. (1990). Functional architecture of basal ganglia circuits: neural substrates of parallel processing. Trends Neurosci. 13, 266–271 1695401 10.1016/0166-2236(90)90107-l

[B3] Alexander G. E. DeLong M. R. Strick P. L. (1986). Parallel organization of functionally segregated circuits linking basal ganglia and cortex. Ann. Rev. Neurosci. 9, 357–381 10.1146/annurev.ne.09.030186.002041 3085570

[B4] Amick M. M. Schendan H. E. Ganis G. Cronin-Golomb A. (2006). Fronto-striatal circuits are necessary for visuomotor transformation: mental rotation in Parkinson's disease. Neuropsychologia 44, 339–349 10.1016/j.neuropsychologia.2005.06.002 16061263

[B5] Angwin A. J. Arnott W. L. Copland D. A. Haire M. P. L. Murdoch B. E. Silburn P. A. (2009). Semantic activation in Parkinson's disease patients on and off levodopa. Cortex 45, 950–959 10.1016/j.cortex.2009.02.012 19356748

[B6] Angwin A. J. Chenery H. J. Copland D. A. Arnott W. L. Murdoch B. E. Silburn P. A. (2004). Dopamine and semantic activation: an investigation of masked direct and indirect priming. J. Int. Neuropsychol. Soc. 10, 15–25 10.1017/S1355617704101033 14751003

[B7] Arévalo A. Baldo J. V. Dronkers N. F. (2012). What do brain lesions tell us about theories of embodied semantics and the human mirror neuron system? Cortex 48, 242–254 10.1016/j.cortex.2010.06.001 20621292 PMC3615255

[B7a] Aziz-Zadeh L. Wilson S. M. Rizzolatti G. Iacoboni M. (2006). Congruent embodied representations for visually presented actions and linguistic phrases describing actions. Curr. Biol. 16, 1818–1823 10.1016/j.cub.2006.07.060 16979559

[B8] Badre D. Wagner A. D. (2007). Left ventrolateral prefrontal cortex and the cognitive control of memory. Neuropsychologia 45, 2883–2901 10.1016/j.neuropsychologia.2007.06.015 17675110

[B9] Bak T. H. Hodges J. R. (2004). The effects of motor neurone disease on language: further evidence. Brain Lang. 89, 354–361 10.1016/S0093-934X(03)00357-2 15068918

[B10] Barrós-Loscertales A. González J. Pulvermüller F. Ventura-Campos N. Bustamante J. C. Costumero V. (2012). Reading salt activates gustatory regions: fMRI evidence for semantic grounding in a novel sensory modality. Cereb. Cortex 22, 2554–2563 10.1093/cercor/bhr324 22123940 PMC4705335

[B11] Barsalou L. W. (2008). Grounded cognition. Annu. Rev. Psychol. 59, 617–645 10.1146/annurev.psych.59.103006.093639 17705682

[B12] Barsalou L. W. Wiemer-Hastings K. (2005). Situating abstract concepts, in Grounding Cognition: The Role of Perception and Action in Memory, Language, and Thought, eds Pecher D. Zwaan R. (New York, NY: Cambridge University Press), 129–163

[B13] Bartels A. Leenders K. L. (2009). Parkinson's disease: the syndrome, the pathogenesis, and pathophysiology. Cortex 45, 915–921 10.1016/j.cortex.2008.11.010 19095226

[B14] Beck A. T. Ward C. H. Mendelson M. Mock J. Erbaugh J. (1961). An inventory for measuring depression. Arch. Gen. Psychiatry 4, 561–571 10.1001/archpsyc.1961.01710120031004 13688369

[B15] Bedny M. McGill M. Thompson-Schill S. L. (2008). Semantic adaptation and competition during word comprehension. Cereb. Cortex 18, 2574–2585 10.1093/cercor/bhn018 18308708 PMC2567420

[B16] Beilock S. L. Lyons I. M. Mattarella-Micke A. Nusbaum H. C. Small S. L. (2008). Sports experience changes the neural processing of action language. Proc. Natl. Acad. Sci. U.S.A. 105, 13269–13273 10.1073/pnas.0803424105 18765806 PMC2527992

[B17] Binder J. R. Desai R. H. (2011). The neurobiology of semantic memory. Trends Cogn. Sci. 15, 527–536 10.1016/j.tics.2011.10.001 22001867 PMC3350748

[B18] Binder J. R. Desai R. H. Graves W. W. Conant L. L. (2009). Where is the semantic system? A critical review and meta-analysis of 120 functional neuroimaging studies. Cereb. Cortex 19, 2767–2796 10.1093/cercor/bhp055 19329570 PMC2774390

[B19] Binney R. J. Embleton K. V. Jeffries E. Parker G. J. M. Lambon Ralph M. A. (2010). The ventral and inferolateral aspects of the anterior temporal temporal lobe are crucial in semantic memory: evidence from a novel direct comparison of distortion-corrected fMRI, rTMS, and semantic dementia. Cereb. Cortex 20, 2728–2738 10.1093/cercor/bhq019 20190005

[B20] Bonner M. F. Peelle J. E. Cook P. A. Grossman M. (2013). Heteromodal conceptual processing in the angular gyrus. Neuroimage 71, 175–186 10.1016/j.neuroimage.2013.01.006 23333416 PMC3594130

[B21] Boulenger V. Metouff L. Thobois S. Broussolle E. Jeannerod M. Nazir T. A. (2008). Word processing in Parkinson's disease is impaired for action verbs but not for concrete nouns. Neuropsychologia 46, 743–756 10.1016/j.neuropsychologia.2007.10.007 18037143

[B22] Buhmann C. Glauche V. Stürenburg H. J. Osner M. Weiller C. Büchel C. (2003). Pharmacologically modulated fMRI—Cortical responsiveness to levodopa in drug-naïve hemiparkinsonian patients. Brain 126, 451–461 10.1093/brain/awg033 12538411

[B23] Calvo-Merino B. Grezes J. Glaser D. E. Passingham R. E. Haggard P. (2006). Seeing or doing? Influence of visual and motor familiarity in action observation. Curr. Biol. 16, 1905–1910 10.1016/j.cub.2006.07.065 17027486

[B24] Candidi M. Sacheli L. M. Mega I. Aglioti S. M. (2013). Somatotopic mapping of piano fingering errors in sensorimotor experts: TMS studies in pianists and visually trained naives. Cereb. Cortex [Epub ahead of print]. 10.1093/cercor/bhs325 23064109

[B25] Carroll J. B. Davies P. Richman B. (1971). The American Heritage Word Frequency Book. New York, NY: American Heritage Publishing Co

[B26] Castiello U. Ansuini C. Bulgheroni M. Scaravilli T. Nicoletti R. (2009). Visuomotor priming effects in Parkinson's disease patients depend on the match between the observed and the executed action. Neuropsychologia 47, 835–842 10.1016/j.neuropsychologia.2008.12.016 19138692

[B27] Catalan M. J. Ishii K. Honda M. Samii A. Hallett M. (1999). A PET study of sequential finger movements of varying length in patients with Parkinson's disease. Brain 122, 483–495 10.1093/brain/122.3.483 10094257

[B28] Chan A. M. Baker J. M. Eskandar E. SSchomer D. Ulbert I. Marinkovic K. (2011). First-pass selectivity for semantic categories in human anteroventral temporal lobe. J. Neurosci. 31, 18119–18129 10.1523/JNEUROSCI.3122-11.2011 22159123 PMC3286838

[B29] Coello Y. Bartolo A. (eds.). (2012). Language and Action in Cognitive Neuroscience. New York, NY: Psychology Press

[B30] Cools R. (2006). Dopaminergic modulation of cognitive function: implications for l-DOPA treatment in Parkinson's disease. Neurosci. Biobehav. Rev. 30, 1–23 10.1016/j.neubiorev.2005.03.024 15935475

[B31] Cooper J. A. Sagar H. J. Jordan N. Harvey N. S. Sullivan E. V. (1991). Cognitive impairment in early, untreated Parkinson's disease and its relationship to motor disability. Brain 114, 2095–2122 10.1093/brain/114.5.2095 1933236

[B32] Copland D. A. (2003). The basal ganglia and semantic engagement: potential insights from semantic priming in individuals with subcortical vascular lesions, Parkinson's disease, and cortical lesions. J. Int. Neuropsychol. Soc. 9, 1041–1052 10.1017/S1355617703970081 14738285

[B33] Copland D. A. McMahon K. Silburn P. A. de Zubicaray G. (2009). Dopaminergic modulation of semantic processing: a 4-T fMRI study with Levodopa. Cereb. Cortex 19, 2651–2658 10.1093/cercor/bhp017 19321651

[B34] Cotelli M. Borroni B. Manenti R. Alberici A. Calabria M. Agosti C. (2006). Action and object naming in frontotemporal dementia, progressive supranuclear palsy, and corticobasal degeneration. Neuropsychology 20, 558–565 10.1037/0894-4105.20.5.558 16938018

[B35] Cross E. S. Hamilton A. F. Grafton S. T. (2006). Building a motor simulation de novo: observation of dance by dancers. Neuroimage 31, 1257–1267 10.1016/j.neuroimage.2006.01.033 16530429 PMC1821082

[B36] Cross E. S. Kraemer D. J. M. Hamilton A. F. Kelley W. M. Grafton S. T. (2009). Sensitivity of the action observation network to physical and observational learning. Cereb. Cortex 19, 315–326 10.1093/cercor/bhn083 18515297 PMC2638791

[B37] Crosson B. Benjamin M. Levy I. (2007). Role of the basal ganglia in language and semantics: supporting cast, in Neural Basis of Semantic Memory, eds Hart J. Jr Kraut M. A. (Cambridge, UK: Cambridge University Place), 219–245

[B38] Cunnington R. Egan G. F. O'Sullivan J. D. Hughes A. J. Bradshaw J. L. Colebatch J. G. (2001). Motor imagery in Parkinson's disease: a PET study. Mov. Disord. 16, 849–857 10.1002/mds.1181 11746614

[B39] Dauer W. Przedborski S. (2003). Parkinson's disease: mechanisms and models. Neuron 39, 889–909 10.1016/S0896-6273(03)00568-3 12971891

[B40] Deen B. McCarthy G. (2010). Reading about the actions of others: biological motion imagery and action congruency influence brain activity. Neuropsychologia 48, 1607–1615 10.1016/j.neuropsychologia.2010.01.028 20138900 PMC3515847

[B41] Dick J. Cantello R. Buruma O. Gioux M. Benecke R. Day B. L. (1987). The Bereitschaftspotential, L-DOPA, and Parkinson's disease. Electroencephalogr. Clin. Neurophysiol. 66, 263–274 2434310 10.1016/0013-4694(87)90075-7

[B42] Dominey P. Decety J. Broussolle E. Chazo G. Jeannerod M. (1995). Motor imagery of a lateralized sequential task is asymmetrically slowed in hemi-Parkinson's patients. Neuropsychologia 33, 727–741 10.1016/0028-3932(95)00008-Q 7675164

[B43] Fahn S. Elton R. L. (1987). The members of the UPDRS Development Committee. Unified Parkinson's disease rating scale, in Recent Developments in Parkinson's Disease, eds Fahn S. Marsden C. D. Goldstein M. Calne D. B. (Florham ark, NJ: McMillan), 153–163

[B44] Fernandino L. Conant L. L. Binder J. R. Blindauer K. Hiner B. Spangler K. (2013). Parkinson's disease disrupts both automatic and controlled processing of action verbs. Brain Lang. [Epub ahead of print]. 10.1016/j.bandl.2012.07.008 22910144 PMC3574625

[B45] Fernandino L. Iacoboni M. (2010). Are cortical motor maps based on body parts or coordinated actions? Implications for embodied semantics. Brain Lang. 112, 44–53 10.1016/j.bandl.2009.02.003 19345405

[B46] Fischer M. Zwaan R. A. (2008). Embodied language: a review of the role of the motor system in language comprehension. Q. J. Exp. Psychol. 61, 825–850 10.1080/17470210701623605 18470815

[B47] Gabrieli J. D. E. Singh J. Stebbins G. T. Goetz C. G. (1996). Reduced working memory span in Parkinson's disease: evidence for the role of a frontostriatal system in working and strategic memory. Neuropsychology 10, 322–332

[B48] Gainotti G. (2012). The format of conceptual representations disrupted in semantic dementia: a position paper. Cortex 48, 521–529 10.1016/j.cortex.2011.06.019 21807363

[B48a] Gerfo E. L. Oliveri M. Torriero S. Salerno S. Koch G. Caltagirone C. (2008). The influence of rTMS over prefrontal and motor areas in a morphological task: grammatical vs. semantic effects. Neuropsychologia 46, 764–770 10.1016/j.neuropsychologia.2007.10.012 18061634

[B49] Gennari S. P. (2012). Representing motion in language comprehension: lessons from neuroimaging. Lang. Linguist. Compass 6, 67–84

[B50] Gibbs R. W. (2006). Embodiment and Cognitive Science. Cambridge, UK: University of Cambridge Press

[B51] Gold B. T. Balota D. A. Jones S. J. Powell D. K. Smith C. D. Andersen A. H. (2006). Dissociation of automatic and strategic lexical-semantics: functional magnetic resonance imaging evidence for differing roles of multiple frontotemporal regions. J. Neurosci. 26, 6523–6532 10.1523/JNEUROSCI.0808-06.2006 16775140 PMC6674026

[B52] González J. Barros-Loscertales A. Pulvermüller F. Meseguer V. Sanjuán A. Belloch V. (2006). Reading cinnamon activates olfactory brain regions. Neuroimage 32, 906–912 10.1016/j.neuroimage.2006.03.037 16651007

[B53] Gotham A. M. Brown R. G. Marsden C. D. (1988). “Frontal” cognitive function in patients with Parkinson's disease “on” and “off” levodopa. Ann. Neurol. 51, 156–164 10.1093/brain/111.2.299 3378138

[B54] Grafton S. T. (2004). Contributions of functional imaging to understanding parkinsonian symptoms. Curr. Opin. Neurobiol. 14, 715–719 10.1016/j.conb.2004.10.010 15582373

[B55] Grossman M. Anderson C. Khan A. Avants B. Elman L. McCluskey L. (2008). Impaired action knowledge in amyotrophic lateral sclerosis. Neurology 71, 1396–1401 10.1212/01.wnl.0000319701.50168.8c 18784377 PMC2676962

[B56] Halsband U. Schmitt J. Weyers M. Binkofski F. Grützner G. Freund H.-J. (2001). Recognition and imitation of pantomimed motor acts after unilateral parietal and premotor lesions: a perspective on apraxia. Neuropsychologia 39, 200–216 10.1016/S0028-3932(00)00088-9 11163376

[B57] Hanakawa T. Fukuyama H. Katsumi Y. Honda M. Shibasaki H. (1999). Enhanced lateral premotor activity during paradoxical gait in Parkinson's disease. Ann. Neurol. 45, 329–336 10072047 10.1002/1531-8249(199903)45:3<329::aid-ana8>3.0.co;2-s

[B58] Haslinger B. Erhard P. Kampfe N. Boecker H. Rummeny E. Schwaiger M. (2001). Event-related functional magnetic resonance imaging in Parkinson's disease before and after levodopa. Brain 124, 558–570 10.1093/brain/124.3.558 11222456

[B59] Hauk O. Shtyrov Y. Pulvermüller F. (2008). The time course of action and action-word comprehension in the human brain as revealed by neurophysiology. J. Physiol. Paris 102, 50–58 10.1016/j.jphysparis.2008.03.013 18485679 PMC2441775

[B60] Helm-Estabrooks N. (2001). Cognitive Linguistic Quick Test. San Antonio, TX: Psychological Corporation

[B61] Helmich R. C. de Lange F. P. Bloem B. R. Toni I. (2007). Cerebral compensation during motor imagery in Parkinson's disease. Neuropsychologia 45, 2201–2215 10.1016/j.neuropsychologia.2007.02.024 17448507

[B63] Hillis A. E. Heidler-Gray J. Newhart M. Chang S. Ken L. Bak T. H. (2006). Naming and comprehension in primary progressive aphasia: the influence of grammatical word class. Aphasiology 20, 246–256

[B64] Hillis A. E. Oh S. Ken L. (2004). Deterioration of naming nouns versus verbs in primary progressive aphasia. Ann. Neurol. 55, 268–275 10.1002/ana.10812 14755731

[B65] Hoehn M. M. Yahr M. D. (1967). Parkinsonism: onset, progression and mortality. Neurology 17, 427–442 6067254 10.1212/wnl.17.5.427

[B66] Hoenig K. Sim E. J. Bochev V. Herrnberger B. Kiefer M. (2008). Conceptual flexibility in the human brain: dynamic recruitment of semantic maps from visual, motor, and motion-related areas. J. Cogn. Neurosci. 20, 1799–1814 10.1162/jocn.2008.20123 18370598

[B67] Hoffman P. Lambon Ralph M. A. (2011). Reverse concreteness effects are not a typical feature of semantic dementia: evidence for the Hub-and-Spoke Model of conceptual representation. Cereb. Cortex 21, 2103–2112 10.1093/cercor/bhq288 21285258

[B68] Hoffman P. Lambon Ralph M. A. (2013). Shapes, scents, and sounds: quantifying the full multisensory basis of conceptual knowledge. Neuropsychologia 51, 14–25 10.1016/j.neuropsychologia.2012.11.009 23159700

[B69] Humphreys G. Newling K. Jennings C. Gennari S. P. (2013). Motion events in language: semantic representations in occipitotemporal cortex. Brain Lang. 125, 94–105 10.1016/j.bandl.2013.01.008 23454619

[B70] Jahanshahi M. Jenkins I. H. Brown R. G. Marsden C. D. Passingham R. E. Brooks D. J. (1995). Self-initiated versus externally triggered movements. I. An investigation using measurement of regional cerebral blood flow with PET and movement-related potentials in normal and Parkinson's disease subjects. Brain 119, 1045–1048 10.1093/brain/119.3.1045 7655888

[B71] Jefferies E. Patterson K. Jones R. W. Lambon Ralph M. A. (2009). Comprehension of concrete and abstract words in semantic dementia. Neuropsychology 23, 492–499 10.1037/a0015452 19586212 PMC2801065

[B72] Jenkins I. H. Fernandez W. Playford E. D. Lees A. J. Frackowiak R. S. J. Passingham R. E. (1992). Impaired activation of the supplementary motor area in Parkinson's disease is reversed when akinesia is treated with apomorphine. Ann. Neurol. 32, 749–757 10.1002/ana.410320608 1471865

[B73] Kable J. W. Lease-Spellmeyer J. Chatterjee A. (2002). Neural substrates of action event knowledge. J. Cogn. Neurosci. 14, 795–805 10.1162/08989290260138681 12167263

[B74] Kable J. W. Kan I. P. Wilson A. Thompson-Schill S. L. Chatterjee A. (2005). Conceptual representations of action in the lateral temporal cortex. J. Cogn. Neurosci. 17, 1855–1870 10.1162/089892905775008625 16356324

[B75] Kemmerer D. Gonzalez-Castillo J. (2010). The Two-Level Theory of verb meaning: an attempt to integrate the semantics of action with the mirror neuron system. Brain Lang. 112, 54–76 10.1016/j.bandl.2008.09.010 18996582 PMC2859696

[B76] Kemmerer D. Gonzalez-Castillo J. Talavage T. Patterson S. Wiley C. (2008). Neuroanatomical distribution of five semantic components of verbs: evidence from fMRI. Brain Lang. 107, 16–43 10.1016/j.bandl.2007.09.003 17977592

[B77] Kemmerer D. Rudrauf D. Manzel K. Tranel D. (2012). Behavioral patterns and lesion sites associated with impaired processing of lexical and conceptual knowledge of actions. Cortex 48, 826–848 10.1016/j.cortex.2010.11.001 21159333 PMC3965329

[B78] Kemmerer D. Tranel D. (2003). A double dissociation between the meanings of action verbs and locative prepositions. Neurocase 9, 421–435 10.1076/neur.9.5.421.16551 14972757

[B79] Kiefer M. Sim E.-J. Herrnberger B. Grothe J. Hoenig K. (2008). The sound of concepts: four markers for a link between auditory and conceptual brain systems. J. Neurosci. 28, 12224–12230 10.1523/JNEUROSCI.3579-08.2008 19020016 PMC6671691

[B80] Kischka U. Kammer T. Maier S. Weisbrod M. Thimm M. Spitzer M. (1996). Dopaminergic modulation of semantic network activation. Neuropsychologia 34, 1107–1113 10.1016/0028-3932(96)00024-3 8904748

[B81] Lambon Ralph M. A. Sage K. Jones R. W. Mayberry E. J. (2010). Coherent concepts are computed in the anterior temporal lobes. Proc. Natl. Acad. Sci. U.S.A. 107, 2717–2722 10.1073/pnas.0907307107 20133780 PMC2823909

[B82] Lange K. W. Paul G. M. Naumann M. Gsell W. (1995). Dopaminergic effects on cognitive performance in patients with Parkinson's disease. J. Neural Transm. Suppl. 46, 423–432 8821078

[B83] Lees A. J. Smith E. (1983). Cognitive deficits in the early stages of Parkinson's disease. Brain 106, 257–270 10.1093/brain/106.2.257 6850270

[B84] Levin B. (1993). English Verb Classes and Alternations. Chicago, IL: University of Chicago Press

[B85] Lewis J. W. (2006). Cortical networks related to human use of tools. Neuroscientist 12, 211–231 10.1177/1073858406288327 16684967

[B86] Lewis S. J. Cools R. Robbins T. W. Dove A. Barker R. A. Owen A. M. (2003). Using executive heterogeneity to explore the nature of working memory deficits in Parkinson's disease. Neuropsychologia 41, 645–654 10.1016/S0028-3932(02)00257-9 12591022

[B87] Lyons I. M. Mattarella-Micke A. Cieslak M. Nusbaum H. C. Small S. L. (2010). The role of personal experience in the neural processing of action-related language. Brain Lang. 112, 214–222 10.1016/j.bandl.2009.05.006 19628276

[B88] Mahon B. Z. Caramazza A. (2005). The orchestration of the sensory-motor systems: clues from neuropsychology. Cogn. Neuropsychol. 22, 480–494 10.1080/02643290442000446 21038262

[B89] Mahon B. Z. Caramazza A. (2008). A critical look at the embodied cognition hypothesis and a new proposal for grounding conceptual content. J. Physiol. Paris 102, 59–70 10.1016/j.jphysparis.2008.03.004 18448316

[B91] Meteyard L. Cuadrado S. R. Bahrami B. Vigliocco G. (2012). Coming of age: a review of embodiment and the neuroscience of semantics. Cortex 48, 788–804 10.1016/j.cortex.2010.11.002 21163473

[B92] Moss H. E. Abdallah S. Fletcher P. C. Bright P. Pilgrim L. K. Acres K. (2005). Selecting among competing alternatives: selection and retrieval in the left inferior frontal gyrus. Cereb. Cortex 15, 1723–1735 10.1093/cercor/bhi049 15728742 PMC3838943

[B93] Moustafa A. A. Sherman S. J. Frank M. J. (2008). A dopaminergic basis for working memory, learning and attentional shifting in Parkinsonism. Neuropsychologia 46, 3144–3156 10.1016/j.neuropsychologia.2008.07.011 18687347

[B94] Murray R. Koenig P. Antani S. McCawley G. Grossman M. (2007). Lexical acquisition in progressive aphasia and frontotemporal dementia. Cogn. Neuropsychol. 24, 48–69 10.1080/02643290600890657 18416483

[B95] Naci L. Taylor K. I. Cusack R. Tyler L. K. (2012). Are the senses enough for sense? Early high-level feedback shapes our comprehension of multisensory objects. Front. Integr. Neurosci. 6:82 10.3389/fnint.2012.00082 23055957 PMC3458237

[B96] Negri G. A. L. Rumiati R. I. Zadini A. Ukmar M. Mahon B. Z. Caramazza A. (2007). What is the role of motor simulation in action and object recognition? Evidence from apraxia. Cogn. Neuropsychol. 24, 795–816 10.1080/02643290701707412 18161497

[B97] Neininger B. Pulvermüller F. (2003). Word-category specific deficits after lesions in the right hemisphere. Neuropsychologia 41, 53–70 10.1016/S0028-3932(02)00126-4 12427565

[B98] Owen A. M. (2004). Cognitive dysfunction in Parkinson's disease: the role of frontostriatal circuitry. Neuroscientist 10, 525–537 10.1177/1073858404266776 15534038

[B99] Papeo L. Hochmann J. R. (2012). A cross-talk between brain-damaged patients and infants on action and language. Neuropsychologia 50, 1222–1234 10.1016/j.neuropsychologia.2012.03.025 22469624

[B100] Papeo L. Negri G. A. L. Zadini A. Rumiati R. I. (2010). Action performance and action-word understanding: evidence of double dissociations in left-damaged patients. Cogn. Neuropsychol. 27, 428–461 10.1080/02643294.2011.570326 21718215

[B101] Patterson K. Nestor P. J. Rogers T. T. (2007). Where do you know what you know? The representation of semantic knowledge in the brain. Nat. Rev. Neurosci. 8, 976–987 10.1038/nrn2277 18026167

[B102] Pazzaglia M. Smania N. Corato E. Aglioti S. M. (2008a). Neural underpinnings of gesture discrimination in patients with limb apraxia. J. Neurosci. 28, 3030–3041 10.1523/JNEUROSCI.5748-07.2008 18354006 PMC6670701

[B103] Pazzaglia M. Pizzamiglio L. Pes E. Aglioti S. M. (2008b). The sound of actions in apraxia. Curr. Biol. 28, 1766–1772 10.1016/j.cub.2008.09.061 19013068

[B104] Pecher D. Boot I. Van Dantzig S. (2011). Abstract concepts: sensory-motor grounding, metaphors, and beyond, in The Psychology of Learning and Motivation, Vol. 54, ed Ross B. (Burlington: Academic Press), 217–248

[B105] Pederzolli A. S. Tivarus M. E. Agrawal P. Kostyk S. K. Thomas K. M. Beversdorf D. Q. (2008). Dopaminergic modulation of semantic priming in Parkinson's disease. Cogn. Behav. Neurol. 21, 134–137 10.1097/WNN.0b013e318185e6f2 18797254 PMC2729120

[B106] Peelen M. V. Caramazza A. (2012). Conceptual object representations in human anterior temporal cortex. J. Neurosci. 32, 15728–15736 10.1523/JNEUROSCI.1953-12.2012 23136412 PMC6621609

[B107] Peelen M. V. Romagno D. Caramazza A. (2013). Is verb selectivity in left posterior temporal cortex related to conceptual action knowledge? J. Cogn. Neurosci. (in press).

[B108] Pierantozzi M. Palmieri M. G. Marciani M. G. Bernardi G. Giacomini P. Stanzione P. (2001). Effect of apomorphine on cortical inhibition in Parkinson's disease patients: a transcranial magnetic stimulation study. Exp. Brain Res. 141, 52–62 10.1007/s002210100839 11685410

[B109] Playford E. D. Jenkins I. H. Passingham R. E. Nutt J. Frackowiak R. S. Brooks D. J. (1992). Impaired mesial frontal and putamen activation in Parkinson's disease: a positron emission tomography study. Ann. Neurol. 32, 151–161 10.1002/ana.410320206 1510355

[B110] Pobric G. Jefferies E. Lambon Ralph M. A. (2009). The role of the anterior temporal lobes in the comprehension of concrete and abstract words: rTMS evidence. Cortex 45, 1104–1110 10.1016/j.cortex.2009.02.006 19303592 PMC2730596

[B111] Pobric G. Jefferies E. Lambon Ralph M. A. (2010). Category-specific versus category-general semantic impairment induced by transcranial magnetic stimulation. Curr. Biol. 20, 964–968 10.1016/j.cub.2010.03.070 20451381 PMC2878637

[B112] Pulvermüller F. (2005). Brain mechanisms linking language and action. Nat. Rev. Neurosci. 6, 576–582 10.1038/nrn1706 15959465

[B113] Pulvermüller F. (2008). Brain embodiment of category-specific semantic memory circuits, in Embodied Grounding: Social, Cognitive, Affective, and Neuroscientific Approaches, eds Semin G. R. Smith E. R. (Cambridge, UK: Cambridge University Press), 71–97

[B114] Pulvermüller F. Cooper-Pye E. Dine C. Hauk O. Nestor P. J. Patterson K. (2009). The word processing deficit in semantic dementia: all categories are equal, but some categories are more equal than others. J. Cogn. Neurosci. 22, 2027–2041 10.1162/jocn.2009.21339 19722916

[B116] Rascol O. Sabatini U. Chollet F. Celsis P. Montastruc J. L. Marc-Vergnes J. P. (1992). Supplementary and primary sensory motor area activity in Parkinson's disease: regional cerebral blood flow changes during finger movements and effects of apomorphine. Arch. Neurol. 49, 144–148 10.1001/archneur.1992.00530260044017 1736846

[B117] Ridding M. C. Inzelberg R. Rothwell J. C. (1995). Changes in excitability of motor cortical circuitry in patients with Parkinson's disease. Ann. Neurol. 37, 181–188 10.1002/ana.410370208 7847860

[B118] Rothwell J. C. Huang Y. Z. (2003). Systems-level studies of movement disorders in dystonia and Parkinson's disease. Curr. Opin. Neurobiol. 13, 691–695 10.1016/j.conb.2003.10.006 14662370

[B119] Rumiati R. I. Zanini S. Vorano L. Shallice T. (2001). A form of ideational apraxia as a selective deficit of contention scheduling. Cogn. Neuropsychol. 18, 617–642 10.1080/02643290126375 20945230

[B120] Sabatini U. Boulanouar K. Fabre N. Martin F. Carel C. Colonnese C. (2000). Cortical motor reorganization in akinetic patients with Parkinson's disease: a functional MRI study. Brain 123, 394–403 10.1093/brain/123.2.394 10648446

[B121] Samuel M. Ceballos-Baumann A. O. Blin J. Uema T. Boecker H. Passingham R. E. (1997). Evidence for lateral premotor and parietal overactivity in Parkinson's disease during sequential and bimanual movements: a PET study. Brain 120, 963–976 10.1093/brain/120.6.963 9217681

[B123] Saygin A. P. (2007). Superior temporal and premotor brain areas necessary for biological motion perception. Brain 130, 2452–2461 10.1093/brain/awm162 17660183

[B124] Saygin A. P. Wilson S. M. Dronkers N. F. Bates E. (2004). Action comprehension in aphasia: linguistic and non-linguistic deficits and their lesion correlates. Neuropsychologia 42, 1788–1804 10.1016/j.neuropsychologia.2004.04.016 15351628

[B125] Scorolli C. Binkofski F. Buccino G. Nicoletti R. Riggio L. Borghi A. M. (2011). Abstract and concrete sentences, embodiment, and languages. Front. Psychol. 2:227 10.3389/fpsyg.2011.00227 21954387 PMC3173827

[B126] Seghier M. L. (2013). The angular gyrus: multiple functions and multiple subdivisions. Neuroscientist 19, 43–61 10.1177/1073858412440596 22547530 PMC4107834

[B127] Semin G. R. Smith E. R. (eds.). (2008). Embodied Grounding: Social, Cognitive, Affective, and Neuroscientific Approaches. Cambridge, UK: Cambridge University Press

[B128] Serino A. De Filippo L. Casavecchia C. Coccia M. Shiffrar M. Ladavas E. (2009). Lesions to the motor system affect action perception. J. Cogn. Neurosci. 22, 413–426 10.1162/jocn.2009.21206 19302003

[B130] Simmons W. K. Barsalou L. W. (2003). The similarity-in-topography principle: reconciling theories of conceptual deficits. Cogn. Neuropsychol. 20, 451–486 10.1080/02643290342000032 20957580

[B131] Simmons W. K. Ramjee V. Beauchamp M. S. McRae K. Martin A. Barsalou L. W. (2007). A common neural substrate for perceiving and knowing about color. Neuropsychologia 45, 2802–2810 10.1016/j.neuropsychologia.2007.05.002 17575989 PMC3596878

[B132] Skipper L. M. Ross L. A. Olson I. R. (2011). Sensory and semantic category subdivisions within the anterior temporal lobes. Neuropsychologia 49, 3419–3429 10.1016/j.neuropsychologia.2011.07.033 21889520 PMC3192293

[B133] Taylor A. E. Saint-Cyr J. A. Lang A. E. (1986). Frontal lobe dysfunction in Parkinson's disease: the cortical focus of neostriatal outflow. Brain 109, 845–883 10.1093/brain/109.5.845 3779372

[B134] Taylor L. J. Zwaan R. A. (2009). Action in cognition: the case of language. Lang. Cogn. 1, 45–58

[B134a] Tettamanti M. Buccino G. Saccuman M. C. Gallese V. Danna M. Scifo P. (2005). Listening to action-related sentences activates fronto-parietal motor circuits. J. Cogn. Neurosci. 17, 273–281 10.1162/0898929053124965 15811239

[B135] Thobois S. Dominey P. Fraix V. Mertens P. Guenot M. Zimmer L. (2002). Effects of subthalamic nucleus stimulation on actual and imagined movement in Parkinson's disease: a PET study. J. Neurol. 249, 1689–1698 10.1007/s00415-002-0906-y 12529791

[B136] Thompson-Schill S. L. D'Esposito M. Kan I. P. (1999). Effects of repetition and competition on activity in left prefrontal cortex during word generation. Neuron 23, 513–522 10.1016/S0896-6273(00)80804-1 10433263

[B137] Thompson-Schill S. L. Swick D. Farah M. J. D'Esposito M. Kan I. P. Knight R. T. (1998). Verb generation in patients with focal frontal lesions: a neuropsychological test of neuroimaging findings. Proc. Natl. Acad. Sci. U.S.A. 26, 14792–14797 10.1073/pnas.95.26.15855 9861060 PMC28134

[B138] Tomasino B. Rumiati R. I. (2013). At the mercy of strategies: the role of motor representations in language understanding. Front. Psychol. 4:27 10.3389/fpsyg.2013.00027 23382722 PMC3562995

[B139] Tranel D. Kemmerer D. Adolphs R. Damasio H. Damasio A. (2003). Neural correlates of conceptual knowledge for actions. Cogn. Neuropsychol. 20, 409–432 10.1080/02643290244000248 20957578

[B140] Tranel D. Manzel K. Asp E. Kemmerer D. (2008). Naming static and dynamic actions: neuropsychological evidence. J. Physiol. Paris 102, 80–94 10.1016/j.jphysparis.2008.03.008 18486456 PMC2519898

[B141] Tranel D. Martin C. Damasio H. Grabowski T. J. Hichwa R. (2005). Effects of noun-verb homonymy on the neural correlates of naming concrete entities and actions. Brain Lang. 92, 288–299 10.1016/j.bandl.2004.01.011 15721961

[B142] Tremblay F. Leonard G. Tremblay L. (2008). Corticomotor facilitation associated with observation and imagery of hand actions is impaired in Parkinson's disease. Exp. Brain Res. 185, 249–257 10.1007/s00221-007-1150-6 17926025

[B143] Ullman M. (2006). Is Broca's area part of a basal ganglia thalamocortical circuit? Cortex 42, 480–485 16881254 10.1016/s0010-9452(08)70382-4

[B144] Van Elk M. van Schie H. T. Hunnius S. Vesper C. Bekkering H. (2008). You'll never crawl alone: neurophysiological evidence for experience-dependent motor resonance in infancy. Neuroimage 43, 808–814 10.1016/j.neuroimage.2008.07.057 18760368

[B145] Visser M. Jefferies E. Lambon Ralph M. A. (2010). Semantic processing in the anterior temporal lobes: a meta-analysis of the functional neuroimaging literature. J. Cogn. Neurosci. 22, 1083–1094 10.1162/jocn.2009.21309 19583477

[B146] Wallentin M. Nielson A. H. Vuust P. Dohn A. Roepstorff A. Lund T. E. (2011). BOLD response to motion verbs in left posterior middle temporal gyrus during story comprehension. Brain Lang. 119, 221–225 10.1016/j.bandl.2011.04.006 21612817

[B147] Wang J. Conder J. A. Blitzer D. N. Shinkareva S. V. (2010). Neural representation of abstract and concrete concepts: a meta-analysis of neuroimaging studies. Hum. Brain Mapp. 31, 1459–1468 10.1002/hbm.20950 20108224 PMC6870700

[B148] Wheatley T. Weisberg J. Beauchamp M. S. Martin A. (2005). Automatic priming of semantically related words reduces activity in the fusiform gyrus. J. Cogn. Neurosci. 17, 1871–1885 10.1162/089892905775008689 16356325

[B149] Willems R. M. Casasanto D. (2011). Flexibility in embodied language understanding. Front. Psychol. 2:116 10.3389/fpsyg.2011.00116 21779264 PMC3132681

[B150] Willems R. M. Hagoort P. (2007). Neural evidence for the interplay between language, gesture, and action: a review. Brain Lang. 101, 278–289 10.1016/j.bandl.2007.03.004 17416411

[B151] Willems R. M. Hagoort P. (2009). Hand preference influences neural correlates of action observation. Brain Res. 1269, 90–104 10.1016/j.brainres.2009.02.057 19272363

[B152] Willems R. Hagoort P. Casasanto D. (2010). Body-specific representations of action verbs: neural evidence from right- and left-handers. Psychol. Sci. 21, 67–74 10.1177/0956797609354072 20424025

[B153] Wilson-Mendenhall C. D. Barrett L. F. Simmons W. K. Barsalou L. W. (2011). Grounding emotion in situated conceptualization. Neuropsychologia 49, 1105–1127 10.1016/j.neuropsychologia.2010.12.032 21192959 PMC3078178

[B154] Wood J. N. Hauser M. D. (2008). Action comprehension in non-human primates: motor simulation or inferential reasoning? Trends Cogn. Sci. 12, 461–465 10.1016/j.tics.2008.08.001 18951832

[B155] Wood J. N. Glynn D. D. Hauser M. D. (2007). The uniquely human capacity to throw evolved from a non-throwing primate: an evolutionary dissociation between action and perception. Biol. Lett. 3, 360–364 10.1098/rsbl.2007.0107 17550878 PMC2390659

